# Stepwise LCST‐Type Phase Separation in Mixtures of Short‐Chain Elastin‐Like Peptides With Minimal Structural Differences

**DOI:** 10.1002/bip.70084

**Published:** 2026-02-13

**Authors:** Naoki Tanaka, Keitaro Suyama, Elissa Mai, Takeru Nose

**Affiliations:** ^1^ Department of Chemistry Faculty and Graduate School of Science, Kyushu University Fukuoka Japan; ^2^ Faculty of Arts and Science, Kyushu University Fukuoka Japan

**Keywords:** coacervation, elastin‐like peptide (ELP), lower critical solution temperature (LCST), peptide materials, phase separation, self‐assembly

## Abstract

Elastin‐like peptides (ELPs) comprise repetitive pentapeptide sequences and exhibit liquid–liquid phase separation through lower critical solution temperature‐type behavior. Their stimuli‐responsive behavior has enabled diverse applications in biomedical and chemical contexts. Although the miscibility and interactions of ELP mixtures have been previously studied, it remains unclear whether mixtures of short‐chain ELPs with minimal differences in intrinsic parameters, such as chain length, can exhibit distinct phase behaviors. In this study, we investigated whether synthetic short‐chain ELPs differing in length by only one or two repeat units (i.e., 5 or 10 residues) could exhibit independent phase transitions in mixed systems. Turbidity measurements of single‐ and two‐component ELP solutions supported by UPLC‐MS analysis revealed stepwise phase transitions upon heating. Our mechanistic analyses revealed that the mixtures undergo a structural transition from polyproline type II helix to β‐sheet or β‐turn structures. In addition, although the mixtures exhibited stepwise phase separation, our results indicate that heterotypic interactions influenced the sequential phase behavior. These findings reveal that even subtle variations in the ELP chain length and architecture can drive distinct phase separation, providing a rational strategy for designing functional, multicomponent, responsive peptide‐based materials.

## Graphical abstract

Small differences in chain‐length or topology induce stepwise phase separation in short‐chain ELP mixtures.

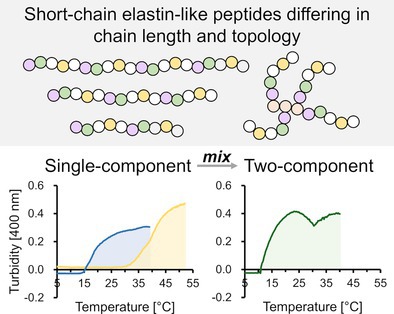

## 1 Introduction

Elastin‐like peptides (ELPs) are synthetic polypeptides derived from the primary sequence of (tropo)elastin. They typically comprise repeating pentapeptide units (VPGXG)_n_, where X represents any amino acid, except proline. ELPs undergo reversible liquid–liquid phase separation (LLPS) via a lower critical solution temperature (LCST)‐type phase behavior, forming a dense, ELP‐rich phase and a dilute phase [1]. The transition temperature (*T*_t_) of ELPs can be tuned by varying their amino acid composition [2, 3, 4], chain length [5, 6], concentration [5, 6], and ionic strength [7, 8], and through chemical modification, including lipidation or conjugation with functional biomolecules [9, 10, 11, 12, 13, 14, 15, 16]. ELPs can be obtained through both recombinant expression and chemical synthesis. Recombinant methods such as recursive directional ligation are particularly suited for obtaining long‐chain ELPs with precisely defined repeat numbers [17]. In contrast, chemical approaches, including solid‐phase peptide synthesis and liquid‐phase peptide synthesis, are useful for synthesizing short‐chain ELPs with desired sequences [18, 19, 20]. These chemical methods enable flexible sequence design through amino acid substitution, insertion, or deletion, enabling fine‐tuning of material properties [21]. As the studies of short ELPs, Renner group focused on behavior of short ELP (one to three repeats) under electrochemical condition [22] and application of single repeat sequence to control the ionomers [23, 24]. In addition to this versatility, short‐chain peptides are also attractive for their ease of synthesis and purification [25]. Collectively, these features make them ideal candidates for engineering LLPS behavior. To date, systematic analyses of the composition and LCST behavior of short‐chain ELPs [26] and studies on their switching behavior [27] have been reported, providing insights into whether short‐chain ELPs can be used as building blocks for smart biomaterials.

The tunable and responsive properties of ELPs have enabled their application in medicine, biotechnology, and chemical engineering, especially where the precise spatiotemporal control of phase separation is required. The stimuli‐responsive self‐assembling property has accelerated their use as drug delivery carriers, protein purification tags, tissue engineering scaffolds, and metal recovery agents [28, 29, 30, 31, 32]. Recently, they have also gained attention as tools for membraneless compartmentalization, in which phase‐separated droplets provide unique physicochemical microenvironments that regulate (bio)chemical reactions [25, 33, 34, 35, 36]. For instance, Chilkoti's group showed that heterotypic condensates of an ELP and mRNA could modulate protein expression [36]. Lampel's group designed a series of minimalistic peptides, some of which contain an ELP motif, and showed these peptides exhibit distinct droplet properties [37]. They further reported that such peptide droplets can regulate a compartmentalized enzymatic reaction, showcasing the potential of short‐chain peptide systems for droplet‐based applications [25].

Despite significant attention being given to the phase behavior of ELPs, how subtle variations in their intrinsic parameters, such as chain length, affect their transitions in multicomponent systems remains poorly understood. Previous studies have shown that ELPs with distinct molecular weights and/or sequences exhibit orthogonal phase behavior. For example, Chilkoti's group observed independent transitions in a binary mixture of long‐chain ELPs that varied both in composition and chain length (*n* = 150 and 160, Δ*n* = 10) [38]. Similarly, van Hest's group revealed that binary mixtures of ELPs of (VPGVG)_n_ only differing in chain length (*n* = 20, 40, and 90; Δ*n* ≥ 20) undergo independent phase transitions upon heating [39]. In contrast, it was shown that two ELPs with highly similar amino acid compositions, or with dissimilar amino acid content but reduced molecular weight in one or both species, tend to form mixed coacervates [40]. These findings indicate that the extent of difference in sequence or length determines whether ELPs phase‐separate independently or form mixed coacervates. However, the effect of minimal differences in chain length, such as one or two repeat units, on the phase behavior of short‐chain ELP mixtures remains unexplored. Understanding how such subtle variations influence LLPS in multicomponent systems is a key step toward fully leveraging the potential of synthetic short‐chain peptide‐based materials.

In this study, we investigated whether synthetic short‐chain ELPs differing in chain length by 1 or 2 repeats (i.e., 5‐residue or 10‐residue differences) could exhibit independent transitions. To this end, short ELP analogs comprise (FPGVG)_n_ [41, 42] (abbreviated as Fn, repetition number *n* = 2–4), which exhibit strong self‐assembly ability with short chain lengths, were prepared, and their self‐assembly ability was studied. First, turbidity measurements were performed on single‐ and two‐component ELP solutions. Turbidity profiles and UPLC‐MS analyses revealed that the ELP mixtures underwent stepwise phase transitions upon heating. Mechanistic insights were obtained using circular dichroism (CD) spectroscopy, dynamic light scattering (DLS) measurements, and molecular dynamics (MD) simulations. The findings showed that even a small difference in the chain length can lead to distinct phase separation. The phase behavior and mechanistic insights into ELPs mixtures revealed in this study support the rational design of synthetic short‐chain peptides as responsive peptide materials with customized phase separation properties.

## 2 Materials & Methods

### 2.1 Chemicals

9‐Fluorenylmethyloxycarbonyl (Fmoc)‐amino acids, Fmoc‐NH‐SAL‐MBHA resin (100–200 mesh), *N*, *N*‐diisopropylethylamine (DIPEA), piperidine, trifluoroacetic acid (TFA), 1‐[(1‐(cyano‐2‐ethoxy‐2‐oxoethylideneaminooxy) dimethylamino‐morpholino)] uronium hexafluorophosphate (COMU), 2‐(1H‐benzotriazole‐1‐yl)‐1,1,3,3‐tetramethyl uronium hexafluorophosphate (HBTU), and OxymaPure were purchased from Watanabe Chemical Industries (Hiroshima, Japan). Triisopropylsilane (TIS) was purchased from Tokyo Chemical Industry (Tokyo, Japan). *N*, *N*‐dimethylformamide (DMF) was purchased from Kanto Chemical (Tokyo, Japan). Water for the experiment was purified using a Milli‐Q EQUATION 7000 (Merck Millipore, Darmstadt, Germany). Other solvents and reagents were obtained from commercial suppliers and used without further purification.

### 2.2 Peptide Synthesis and Purification

The linear peptides H‐(FPGVG)_n_‐NH_2_ (Fn, *n* = 1–4) and Fmoc‐[α‐E(OH)]_3_‐FPGVG‐NH_2_ (a backbone peptide for the synthesis of a branched ELP) were obtained by solid‐phase synthesis based on Fmoc strategy with HBTU/OxymaPure manually or on a CSBio II, automated peptide synthesizer (Menlo Park, CA). After the peptide chain elongation, cleavage was performed with a solution of 95% TFA, 2.5% TIS, and 2.5% H_2_O. Then, for Fn, the TFA solution was purified with a Sep‐Pak cartridge (C18, 10 g) and HPLC followed by lyophilization to give target Fn as a colorless solid. For Fmoc‐[α‐E(OH)]_3_‐FPGVG‐NH_2_, cold diethyl ether was added to the TFA solution, and the precipitate was dried under vacuum to give the oligopeptide as a colorless solid.

The branched ELP was obtained by a method similar to that previously reported as follows [43]. Fmoc‐[E(OH)]_3_‐FPGVG‐NH_2_ (0.03 mmol, 1 eq), F1 (0.108 mmol, 3.6 eq), COMU (0.18 mmol, 6 eq), and DIPEA (0.216 mmol, 7.2 eq) were dissolved in 400 μL of DMF and the solution was stirred at room temperature for a day. Then, 400 μL of water was added, and the solution was stirred for 1 h. Piperidine was added to the solution to give 20% (v/v) piperidine solution and kept stirred for 2 h. The reaction mixture was filtered and applied to Sep‐Pak (C18, 2 g) for pre‐purification, and purified with RP‐HPLC (JASCO PU‐4180 equipped with UV‐4075, JASCO, Tokyo, Japan) using a C8 column (COSMOSIL 5C8‐AR‐300 Packed Column, 20 mmI.D. × 150 mm, Nacalai Tesque Inc., Kyoto, Japan). The solvent system for RP‐HPLC consisted of 0.1% TFA aqueous solution (v/v, solvent A) and mixture of 80% acetonitrile and 20% solvent A (v/v, solvent B). The purified fractions were then evaporated and lyophilized to give the target branched ELP, [α‐E(F1)]_3_‐F1 (H‐[E(FPGVG‐NH_2_)]_3_‐FPGVG‐NH_2_), as a colorless solid. For brevity, this peptide is denoted as 4F1 hereafter.

The obtained peptides were analyzed for their purity by ACQUITY UPLC H‐Class (Waters Co., Milford, MA) equipped with an ACQUITY UPLC BEH C‐18 column (50 mm, Waters Co.). The solvent system for UPLC consisted of 0.1% formic acid aqueous solution (v/v) and 0.1% formic acid in acetonitrile (v/v).

### 2.3 Turbidity Measurement

The LCST‐type phase behavior of ELPs was analyzed using a JASCO V‐660 spectral photometer (JASCO, Tokyo, Japan). The ELPs were dissolved either as single‐component solutions or as mixture solutions in phosphate buffer (pH 7.4, 27.4 mM Na_2_HPO_4_, 17.8 mM NaH_2_PO_4_) containing NaCl (1 M or 3 M NaCl). NaCl in this concentration is used to induce LCST‐type phase behavior for the inverse transition cycling, a method for purifying proteins based on the LCST behavior of ELPs [24]. Turbidity at 400 nm was traced while increasing or decreasing the temperature at a rate of 0.5°C/min. For single‐component ELP solutions, the transition temperature (*T*_t_) was defined as a temperature at which the turbidity reaches half of its maximum value during heating. Measurements were performed at least three times.

### 2.4 UPLC‐MS Analysis

UPLC‐MS was used to quantify ELPs remaining in the dilute phase after LCST‐type phase separation. Peak areas of ELPs were analyzed in the following samples to determine the percentages of remaining ELPs in the supernatant after incubation: (1) the mixture solution at 5°C, (2) the supernatant after incubation at 25°C, and (3) the supernatant after incubation at 50°C. To prepare samples (2) and (3), the mixture solution was incubated at either 25°C or 50°C for 30 min, followed by centrifugation at 14,000 rpm for 2 min at 25°C. The resulting supernatant was analyzed using UPLC‐MS. The percentage of remaining ELPs in the supernatant after incubation at 25°C and 50°C was calculated using the following equations:

$$\begin{array}{c} \text{Peptides remained}\;\mathrm{at}\;25^{{}^{\circ}}C\;\left(\%\right)=100\times \text{peak area in}\;\left(2\right)/\\ \;\text{peak area in}\;\left(1\right) \end{array}$$

$$\begin{array}{c} \text{Peptides remained}\;\mathrm{at}\;50^{{}^{\circ}}C\;\left(\%\right)=100\times \text{peak area in}\;\left(3\right)/\\ \;\text{peak area in}\;\left(1\right) \end{array}$$

Measurements were performed at least three times.

### 2.5 Optical Microscopy

The ELP aggregates were observed using a Leica DM IL LED microscope (Leica Microsystems CMS GmbH, Wetzlar, Germany) equipped with a HI PLAN 40 × oil objective (Leica Microsystems CMS GmbH) and an HC PLAN 10 × eyepiece (Leica Microsystems CMS GmbH). Single‐component and two‐component solutions were prepared as described in turbidity measurement. Sample imaging was performed at 5°C, 25°C, and 50°C using a Thermo Plate TP‐CHSQM (Tokai Hit, Shizuoka, Japan). White balance was adjusted on Leica Application Suite X (Leica Microsystems CMS GmbH).

### 2.6 DLS Measurement

The particle size distribution of short‐chain ELPs was analyzed in phosphate buffer with 1 M or 3 M NaCl (pH 7.4, 27.4 mM Na_2_HPO_4_, 17.8 mM NaH_2_PO_4_) using DLS with a Zetasizer Nano ZS (Malvern Instruments, Worcestershire, UK). DLS analysis was performed across 5°C–50°C. Before measurements, the cell holder was purged with nitrogen gas. The measurement duration was selected automatically. The parameter dataset “protein” (dataset: refractive index, 1.450; absorption, 0.001) was used as the material parameter, and the parameter dataset “water” (dataset: refractive index, 1.330; viscosity, 0.8872) was chosen as the dispersant parameter. Attenuation was selected automatically. Measurements were performed at least three times. The autocorrelation curves are shown in Figure S11.

### 2.7 CD Measurement

Circular dichroism (CD) measurements were carried out using a J‐725 spectropolarimeter (JASCO) in a cuvette with a 1.0 mm path‐length. The ELPs were dissolved in filtered phosphate buffer (pH 7.4, 27.4 mM Na_2_HPO_4_, 17.8 mM NaH_2_PO_4_). The final concentrations were as follows: F4 + F3 (F4 0.0210 mg/mL and F3 0.0790 mg/mL, 0.1000 mg/mL in total), F4+ 4F1 (F4 0.0210 mg/mL and 4F1 0.0508 mg/mL, 0.0718 mg/mL in total), F4 (0.021 mg/mL), F3 (0.0790 mg/mL), and 4F1 (0.0508 mg/mL). The mixture concentrations were adjusted to maintain the same ratio as used in turbidimetry. Under these conditions, apparent aggregation behaviors were not observed. Spectra were obtained from 190 to 260 nm at temperatures ranging from 5°C to 55°C with 10°C intervals. Each measurement was performed after 2 min of equilibration at the target temperature. After subtracting the background spectra, Savitzky–Golay filters were applied to smooth all spectra. Theoretical spectra of binary mixtures were calculated in terms of mean residue ellipticity (*θ*, deg cm^3^ dmol^−1^) or CD signal (mdeg) using the following equations:

(1) $$\theta_{\text{calcd}}=\left(C_{A}\theta_{A}+C_{B}\theta_{B}\right)/\left(C_{A}+C_{B}\right)$$

(2) $${\mathrm{CD}}_{\text{calcd}}={\mathrm{CD}}_{A}+{\mathrm{CD}}_{B}$$

where *C*_A_ and *C*_B_ are the residue molar concentrations of peptide A and B, respectively; *θ*_A_ and *θ*_B_ are the experimentally obtained *θ* of peptide A and B; and CD_A_ and CD_B_ are their corresponding CD signals in mdeg.

### 2.8 Molecular Dynamics (MD) Simulation

MD simulations of a single molecule were performed for F4, F3, F2, and 4F1 using a DELL PRECISION T3610 workstation (Dell Inc. Round Rock, TX, USA). The initial conformations of peptides were generated by Discovery studio 4.0 software (Dassault Systemes BIOVIA, San Diego, CA, USA). The MD simulations were performed in GROMACS 2019 with AMBER99SB‐ILDN force field and TIP3P explicit solvent model [44]. The trajectories of these peptides were obtained at simulation temperature of 278, 298, and 323 K (5°C, 25°C, and 50°C) for 50 ns for F4, F3, and 4F1, and for 100 ns for F2. Then, the radius of gyration (Rg), solvent accessible surface areas (SASA), the number of intramolecular hydrogen bonds (nPP), and the number of hydrogen bonds between water and peptide (nPW) were analyzed by omitting the first 10 ns. The detailed calculation protocols are described in the Supporting Information.

## 3 Results & Discussion

### 3.1 Turbidity Profiles

**FIGURE 1:**
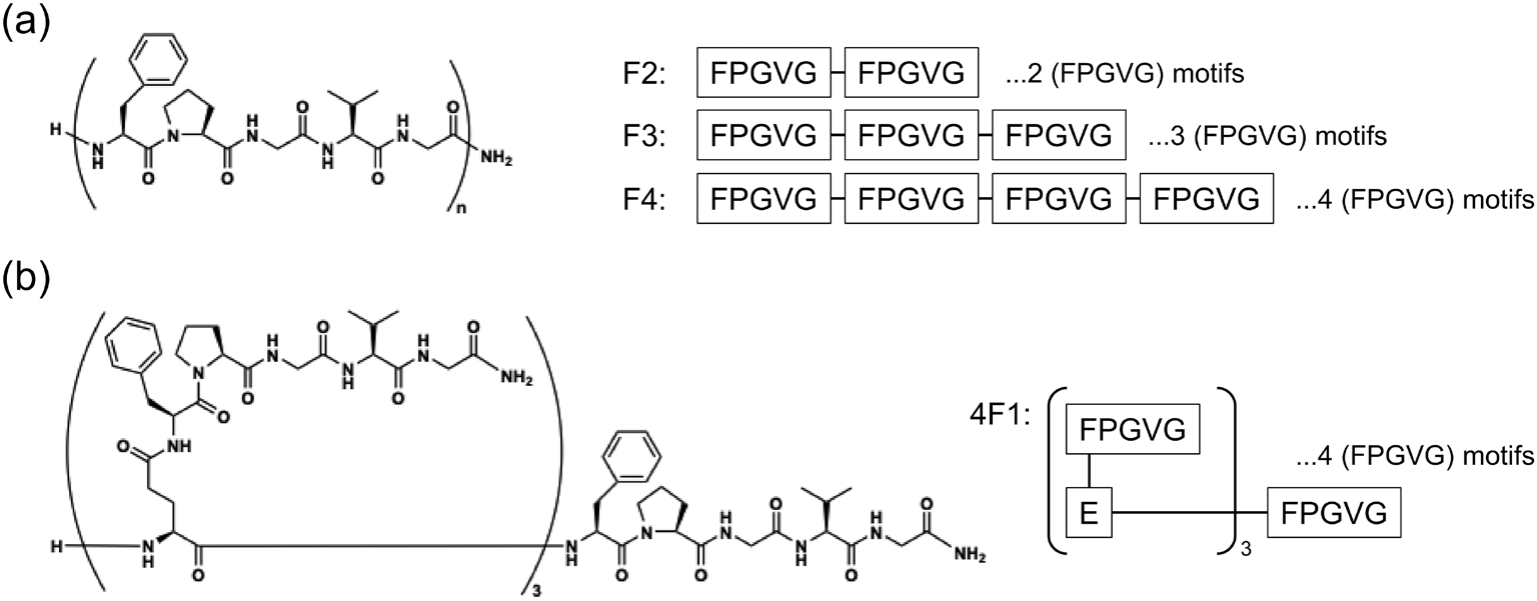
Chemical structures of ELPs. (a) Linear short‐chain ELPs (*n* = 2, 3, or 4), and (b) branched short‐chain ELP [α‐E(F1)]_3_‐F1 (4F1). Linear ELPs (FPGVG)_n_ are abbreviated as Fn, where *n* represents the number of repetitions. Branched ELP is abbreviated as 4F1 because it contains four F1 units (four FPGVG chains).

The target linear ELPs were successfully obtained using solid‐phase peptide synthesis (Figure 1a, Figure S1, and Table S1). The linear ELPs (FPGVG)_n_ are abbreviated as Fn, where *n* represents the number of repetitions. Linear ELPs F4, F3, and F2 are comprised of 20, 15, and 10 amino acid residues, respectively. In other words, F4, F3, and F2 share the same amino acid composition, but differ in chain length by one or two repeats (i.e., 5‐ or 10‐residue differences). The branched ELP ([α‐E(F1)]_3_‐F1, Figure 1b) was also successfully obtained by condensing F1 chains (pentapeptide FPGVG units) and the backbone peptide. This peptide is abbreviated as 4F1 because it contains four chains of F1. The number of pentapeptide units in 4F1 was the same as that in F4.

**FIGURE 2:**
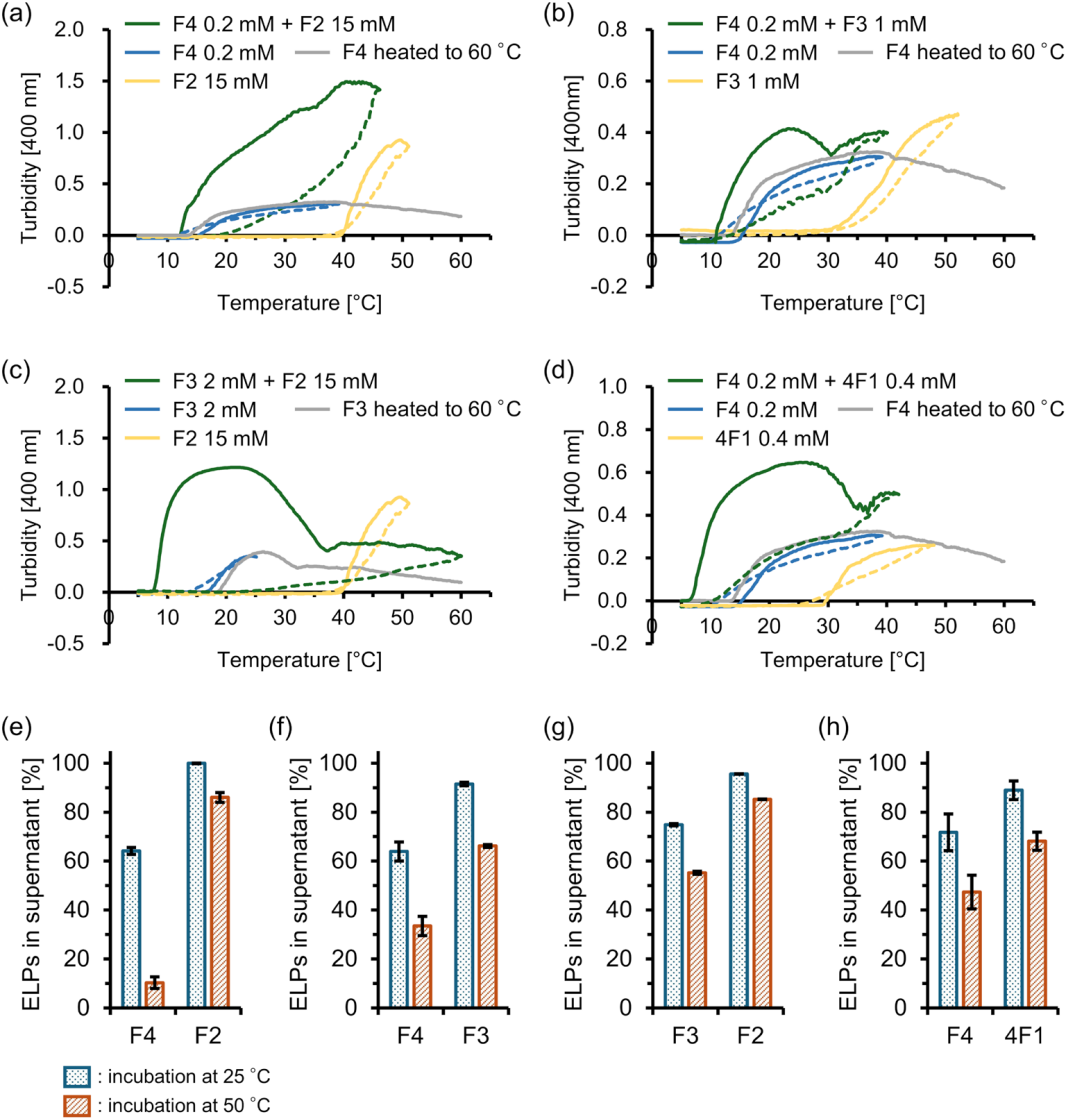
Turbidity profiles and quantification of ELPs in the dilute phase. Turbidity profiles of (a) F4, F2, and F4 + F2, (b) F4, F3, and F4 + F3, (c) F3, F2, and F3 + F2, and (d) F4, 4F1, and F4 + 4F1. ELPs remained in the supernatant of mixture after incubation at 25°C and 50°C for (e) F4 and F2, (f) F4 and F3, (g) F3 and F2, and (h) F4 and 4F1. Mixture samples were prepared as follows: (a, e) 0.2 mM F4 and 15 mM F2 in phosphate buffer with 3 M NaCl, (b, f) 0.2 mM F4 and 1 mM F3 in phosphate buffer with 3 M NaCl, (c, g) 2 mM F3 and 15 mM F2 in phosphate buffer with 3 M NaCl, and (d, h) 0.2 mM F4 and 0.4 mM 4F1 in phosphate buffer with 3 M NaCl.

Turbidity measurements were conducted to investigate phase behavior. First, the phase behavior of each ELP was investigated. Each ELP showed LCST‐type behavior with concentration‐dependent *T*_t_ values following the equation *T*_t_ = *a* log (*C*_ELP_) + *b*, where *C*_ELP_ is the ELP concentration, and *a* and *b* are constant values (Figures S2 and S3, Table S2) [5]. In phosphate buffer supplemented with 3 M NaCl, F4 at 0.2 mM exhibited LCST‐type behavior with a *T*_t_ value of approximately 18°C, and F2 at 15 mM and 4F1 at 0.4 mM showed LCST‐type behavior at approximately 50°C and 34°C, respectively (Figure 2a,c,d). F3 at 1 and 2 mM also showed LCST‐type behavior at approximately 40°C and 21°C, respectively (Figure 2b,c). In addition, it was confirmed that the individual F4 and F3 exhibited a single turbidity increase when heated to 60°C, followed by a decrease in turbidity at high temperatures, which could be attributed to the precipitation of Fn coacervates.

Next, the ELP mixtures were subjected to turbidimetry. Based on the turbidimetry results for individual ELPs, the peptide concentration conditions for a mixture of ELPs in phosphate buffer with 3 M NaCl were determined. Concentration conditions were set such that (i) each peptide exhibited a measurable turbidity increase and (ii) the *T*_t_ values of the components differed by at least 10°C, enabling the differentiation of individual contributions within a mixture. In addition, to investigate the effect of minimal differences in chain length on phase behavior of short‐chain ELP, the difference in repeating motifs (Δ*n*) of the ELPs mixed was kept to a minimum (Δ*n* = 0–2). The specific experimental conditions were as follows: (i) F4 + F2, F4 (0.2 mM) and F2 (15 mM), Δ*n* = 2; (ii) F4 + F3, F4 (0.2 mM) and F3 (1 mM), Δ*n* = 1; (iii) F3 + F2, F3 (2 mM) and F2 (15 mM), Δ*n* = 1; (iv) F4 + 4F1, F4 (0.2 mM) and 4F1 (0.4 mM), Δ*n* = 0. ELP mixtures were also assessed in phosphate buffer with 1 M NaCl, and peptide concentrations were determined as follows: (v) F4 + F3, F4 (3 mM) and F3 (10 mM); and (vi) F4 + 4F1, F4 (3 mM) and 4F1 (5 mM). For each ELP mixture pair denoted as “A + B”, the *T*_t_ value of peptide A as measured in its single‐component sample was set to be lower than that of peptide B under the respective concentration condition.

A mixed solution of F4 and F2, which was designed to have the same ratio of pentapeptide units as (VPGVG)_40_ and (VPGVG)_20_ in a previous study [37], was examined. Upon heating, the F4 + F2 mixture solution exhibited a two‐step turbidity increase, the first beginning at approximately 10°C and the second at approximately 35°C (Figure 2a). This turbidity profile indicates that the two ELP underwent stepwise phase separation, with F4 undergoing phase separation at a lower temperature and F2 at a higher temperature. Notably, the onset temperature of turbidity increase for the mixture was lower than that of the individually prepared F4, possibly because of the higher total ELP concentration relative to the single‐component system, and heterotypic interactions between F4 and F2 may have facilitated the first transition at lower temperatures. In addition, the turbidity of F4 + F2 was higher than that of F4. In this study, the turbidity values were not normalized because the differences in turbidity potentially reflect variations in droplet properties (such as size, number, and morphology). The enhanced turbidity observed in the mixture suggests heterotypic interactions between F4 and F2 during phase separation, potentially altering the droplet properties, even though phase separation proceeds in a stepwise manner. Furthermore, the second turbidity increase (primarily attributed to the phase transition of F2) began at a lower temperature than that of individually prepared F2. This shift could be due to the presence of phase‐separated F4 droplets, which reduce the effective volume available for F2 and increase its apparent concentration, thereby promoting phase transition. Upon cooling, turbidity decreased in a single step, in contrast to the two‐step increase observed during heating. This hysteresis was attributed to the different kinetic pathways governing self‐assembly during heating and dissociation during cooling. The phase transition during heating occurs in a stepwise manner, where heterotypic interactions between components promote cooperative aggregation, whereas cooling results in the rapid dissolution of components and a more monophasic turbidity change. Therefore, kinetic effects contributed significantly to the aggregation behavior of the two‐component system in this study. Simultaneously, the overall temperature‐dependent behavior (including the measurable *T*_t_ and sigmoidal heating profiles) in the two‐component system can still be described in the thermodynamic framework.

We then examined the phase behavior of the additional two‐component samples (Figure 2b–d). The F4 + F3 and F3 + F2 mixtures were designed such that the ELPs in each pair differed by one pentapeptide unit in chain‐length (Δ*n* = 1). F4 + 4F1 was designed such that each ELP contained the same number of pentapeptides FPGVG and the same net charge (+1), but differed in topology, being either linear or branched. Notably, their chemical compositions were not strictly identical owing to the branched linkage of the triGlu backbone. The mixture samples showed an increase in turbidity at lower temperatures than the single‐component peptide A of each pair, similar to the results for the F4 + F2 mixed solution. Upon further heating, F4 + F3 and F4 + 4F1 exhibited a two‐step increase in turbidity. In F3 + F2, although the two‐step turbidity increase was not clear upon heating to 60°C, the turbidity decrease was relatively gradual and the turbidity values were higher than those of the F3‐only sample. The onset temperatures of the second turbidity increase in F4 + F3 and F4 + 4F1 were close to or higher than those of single‐component peptide B, in contrast to F4 + F2. These differences can be explained by the partitioning of peptide B to peptide A droplets or the co‐assembly of the pairs at lower temperature ranges (10°C–30°C). When the heterotypic interactions between peptides A and B are favorable and result in partitioning or co‐assembly, peptide B is depleted in the dilute phase. The resulting low apparent concentration of peptide B led to a second phase separation at a higher temperature.

The two‐step phase separation was further validated using UPLC‐MS analysis. Samples heated to 25°C or 50°C were centrifuged and analyzed, and their peak areas were compared to those at 5°C. Because the volume of the condensed phase was too small to collect, only the dilute phase was analyzed (Figure S4). Based on the turbidimetry, it was expected that at 25°C, peptide A would be partially reduced and peptide B would remain in the supernatant, whereas at 50°C, both ELPs would be depleted. For F4 + F2, the UPLC‐MS results showed that at 25°C, the amount of F4 in the supernatant decreased, whereas that of F2 remained almost unchanged (Figure 2e). At 50°C, F4 was further depleted and F2 was also partially removed. The other pairs (F4 + F3, F3 + F2, and F4 + 4F1) showed similar trends (Figure 2f–h): the amount of peptide A selectively decreased at 25°C and further decreased at 50°C, and peptide B partially decreased at 25°C and further decreased at 50°C. These results reveal that peptide A selectively phase‐separated at 25°C, and the phase separation of peptide B occurred upon further heating.

**FIGURE 3:**
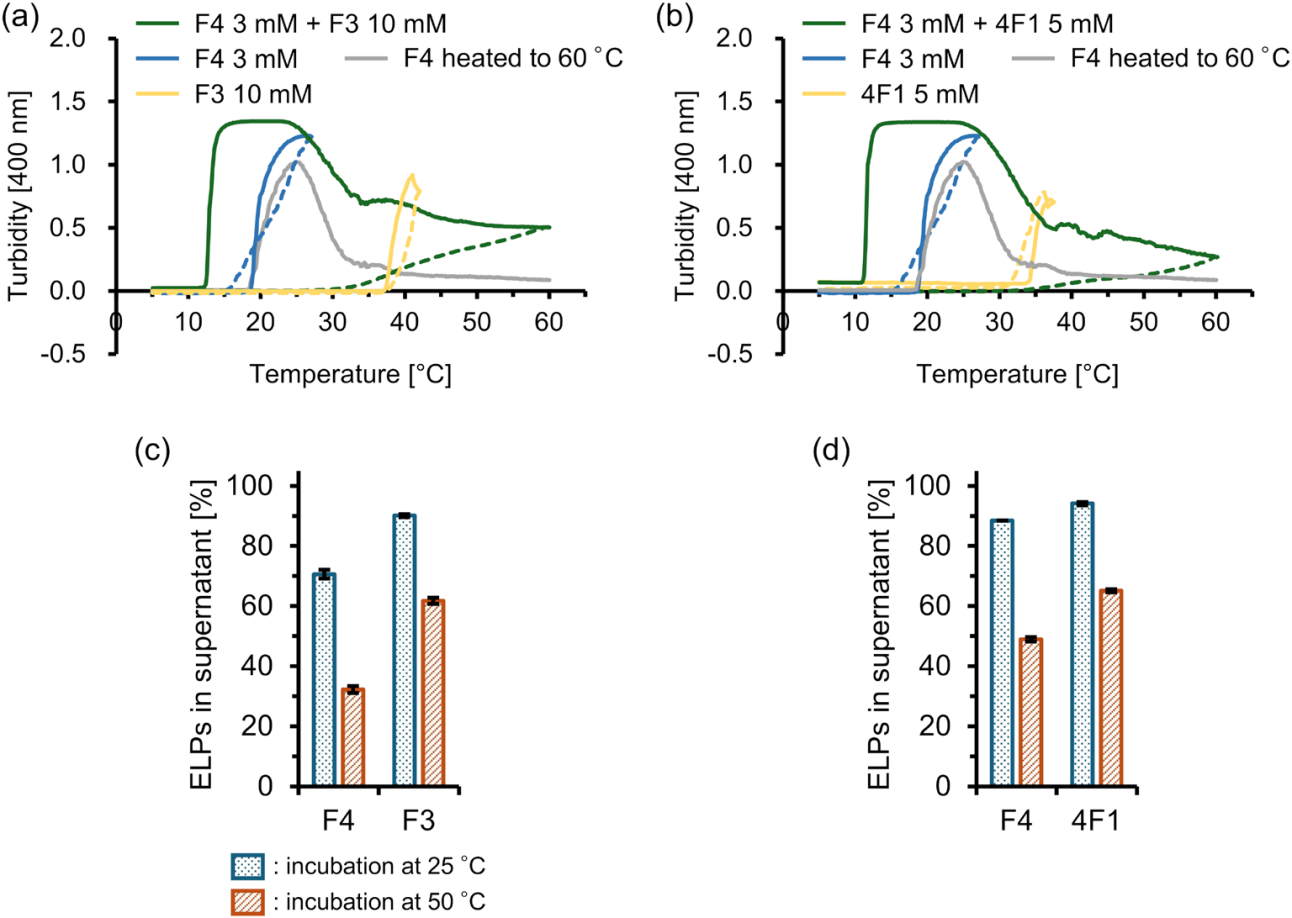
Turbidity profiles and quantification of ELPs in the dilute phase under a reduced ionic strength. Turbidity profiles of (a) F4, F3, and F4 + F3, and (b) F4, 4F1, and F4 + 4F1. ELPs remained in the supernatant after incubation at 25°C and 50°C for (c) F4 and F3, and (d) F4 and 4F1. Mixture samples were prepared as follows: (a, c) 3 mM F4 and 10 mM F3 in phosphate buffer with 1 M NaCl, and (b, d) 3 mM F4 and 5 mM 4F1 in phosphate buffer with 1 M NaCl.

In addition, two‐component samples in a phosphate buffer with reduced ionic strength (phosphate buffer with 1 M NaCl) were investigated for temperature‐responsive behavior. Under these conditions, single‐component F4 at 3 mM exhibited LCST‐type behavior, with *T*_t_ values of 20°C (Figure 3a,b). F3 at 10 mM and 4F1 at 5 mM also exhibited LCST‐type behavior at approximately 36°C and 33°C, respectively (Figure 3a,b). Again, it was confirmed that the single‐component F4 exhibited a single turbidity increase when heated to 60°C. The decrease in turbidity at high temperatures could be attributed to the precipitation of F4 coacervates. The F4 + F3 mixture showed an increase in turbidity at approximately 12°C, which is lower than the *T*_t_ of F4 alone (Figure 3a). Although no distinct two‐step turbidity increase was observed upon heating to 60°C, the turbidity decrease was relatively gradual, and the turbidity values were higher than those of the F4‐only sample. The F4 + 4F1 mixture showed a similar turbidity trend, with turbidity increase at approximately 10°C followed by a slow turbidity decrease upon further heating (Figure 3b). The turbidity increases at approximately 10°C is again likely because of the higher total ELP concentration, which facilitated the transition at a low temperature. Although these two pairs did not exhibit an apparent two‐step turbidity increase, the decreased yet stable turbidity observed at 25°C–60°C may be because phase transitions of peptide B of the pairs occurred at 30°C–40°C in each mixture. The UPLC‐MS analysis revealed that the amount of ELPs remaining in the supernatant decreased at 25°C, but with a more significant reduction in peptide A (F4) in F4 + F3 and F4 + 4F1 (Figure 3c,d). At 50°C, F4 was further depleted and the partner ELP also decreased. These results indicate that in these mixtures, one ELP underwent phase separation at a lower temperature with the partner ELP partially partitioned or co‐assembled, whereas the partner ELP gradually phase‐separated upon further heating. This stepwise transition can be attributed to the distinct depletion kinetics of each component, indicating that the interactions between ELP pairs involve specific heterotypic affinities.

### 3.2 Droplet Morphology

The higher turbidity observed in the mixture indicates that it contained more efficient scatterers at high temperatures than the single‐component solutions. This result indicates that the presence of another peptide may modify the aggregate structure or increase the number of efficient scatterers (at 400 nm) through intermolecular heterotypic or homotypic interactions. Based on these results, imaging using optical microscopy and size distribution analysis using DLS were performed.

**FIGURE 4:**
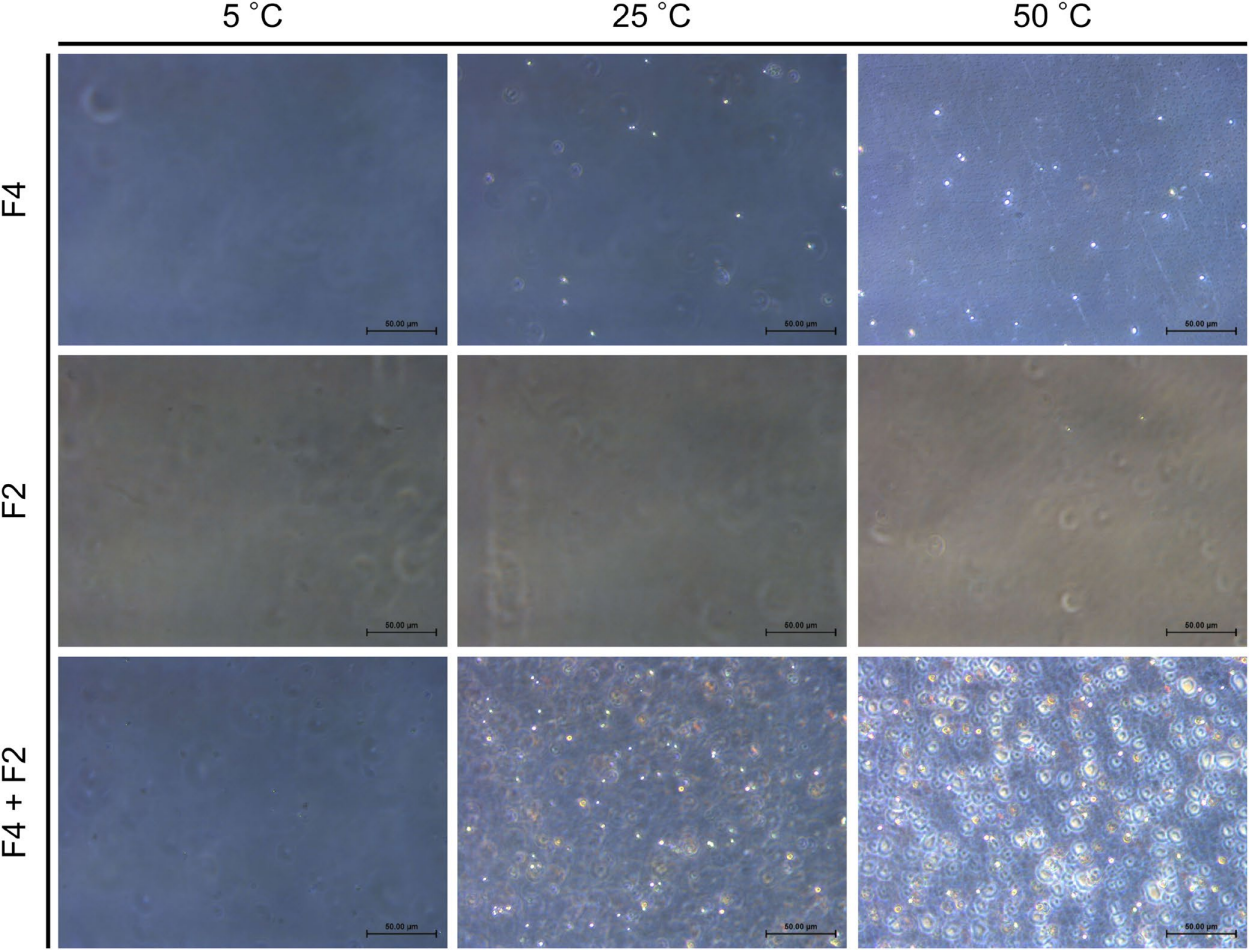
Microscopy images of F4, F2, and F4 + F2 in phosphate buffer supplemented with 3 M NaCl. For single‐component samples, F4 and F2 were dissolved at 0.2 mM and 15 mM, respectively. In the mixed sample (F4 + F2), F4 and F2 were dissolved at 0.2 mM and 15 mM, respectively. Pictures were taken after 2 min of equilibration at the target temperatures. Scale bars indicate 50 μm.

Bright field microscopy images showed that the individually prepared F4 in phosphate buffer supplemented with 3 M NaCl formed spherical droplets and precipitated onto the bottom surface of the slide at 25°C and 50°C (Figure 4). Similarly, spherical droplets of F2 were observed at 50°C. In the F4 + F2 sample, the number of droplets was apparently larger than that of the F4‐only samples at 25°C, and precipitated droplets merged into larger droplets on the bottom surface of the slide at 50°C. A greater number of droplets resulted in higher turbidity in the mixed sample. Similar trends were observed for F4 + F3, F3 + F2, and F4 + 4F1 (Figures S5–S7). The individually prepared F4 in phosphate buffer supplemented with 1 M NaCl also formed spherical droplets and precipitated onto the bottom surface of the slide at 25°C and 50°C (Figure S8). For F4 + F3 and F4 + 4F1, droplets rapidly precipitated onto the bottom of the slide at 25°C, forming larger precipitates compared to the individually prepared F4. At 50°C, the precipitates became larger and denser due to the newly precipitated droplets.

**FIGURE 5:**
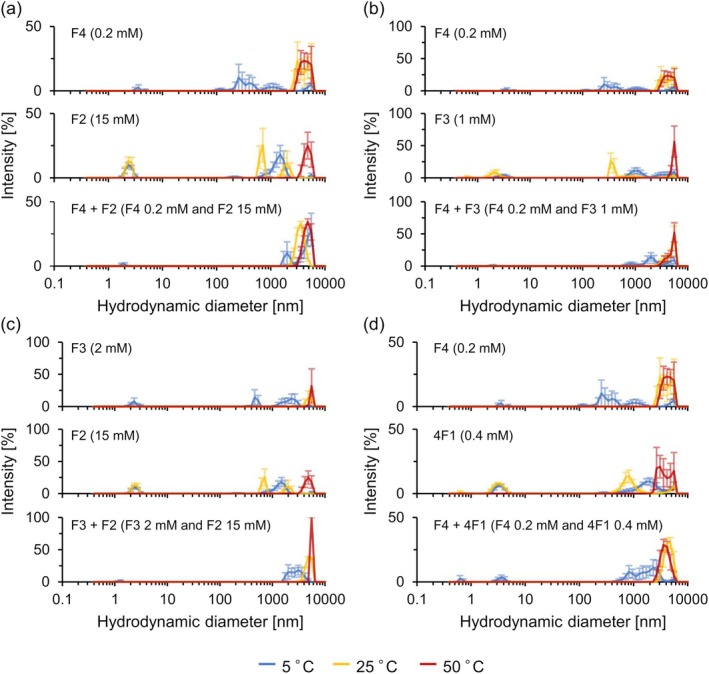
Hydrodynamic diameter obtained by DLS measurement. (a) F4, F2, and F4 + F2, (b) F4, F3, and F4 + F3, (c) F3, F2, and F3 + F2, and (d) F4, 4F1, and F4 + 4F1. Samples were prepared in phosphate buffer supplemented with 3 M NaCl. Results are shown in intensity (%) with standard errors.

Next, DLS measurements were performed to investigate the changes in the particle size distribution with temperature. Generally, DLS measurements have well‐known limitations in accurately determining particle sizes above 1 μm because the Brownian motion of such large particles becomes too slow to yield reliable correlation data, and additional effects such as directional scattering and sedimentation further compromise the measurement. Therefore, in this study, DLS measurements were performed to compare the relative size changes and the onset of aggregation across different peptide combinations and temperature conditions, rather than obtaining absolute particle diameters. DLS measurements of the individual components (F4, F3, F2, and 4F1) and their mixtures revealed that the aggregate size increased with heating (Figure 5a–d, Figure S9). Figure 5 shows the DLS results for the scattering intensity (%), whereas Figure S9 shows them as numbers (%). In addition, the size distribution of each single‐component sample is not conserved in the binary mixtures, particularly at low temperatures. This result indicates that when mixed, the peptides interact through both homotypic and heterotypic interactions, resulting in aggregation states distinct from those of the individual components. Furthermore, temperature‐dependent changes in the scattering intensities varied among the samples (Figure S10). In the single‐component samples, scattering intensity increased when the temperature was higher than the *T*_t_ of each peptide (note that in each pair of peptide A and B, the single‐component peptide A has a *T*_t_ below 25°C, while the peptide B has a *T*_t_ above 25°C under the measurement conditions). In contrast, in the binary mixtures, the trend of the temperature‐dependent changes in the scattering intensity differed among the samples. Microscopy and DLS results revealed that the aggregation properties of the mixtures were affected by interpeptide interactions, which may result in distinct aggregate sizes, distributions, and number densities.

### 3.3 Mechanistic Insights

Previously, we investigated the structural changes in linear and branched short ELPs in response to temperature changes using CD measurements [21, 41, 42, 43]. Both H‐(FPGVG)_n_‐NH_2_ and [α‐E(F1)]_n_‐F1 showed a negative peak at approximately 200 nm and a small positive peak at 225 nm at low temperatures, indicating that these peptides adopted a polyproline type II (PPII)‐like helical structure at low temperatures [45]. Upon heating, the intensity of these peaks decreased, and a small shoulder peak appeared at 210 nm, revealing a structural transition to conformations rich in β‐turns or β‐sheet structures at high temperatures. In this study, CD measurements were performed on single‐ and two‐component samples to investigate whether the secondary structural characteristics of these short ELPs were affected by the presence of other peptides. Among the mixed samples, F4 + F3 and F4 + 4F1 were selected for analysis. F4 and F3 differ only in their chain length by a pentapeptide unit, whereas F4 and 4F1 have the same number of units, but differ in their molecular architecture. The CD data (mdeg, shown in Figures S12 and S13) were converted to mean residue ellipticity (*θ*), as shown in Figure 5 and Figure S14.

**FIGURE 6:**
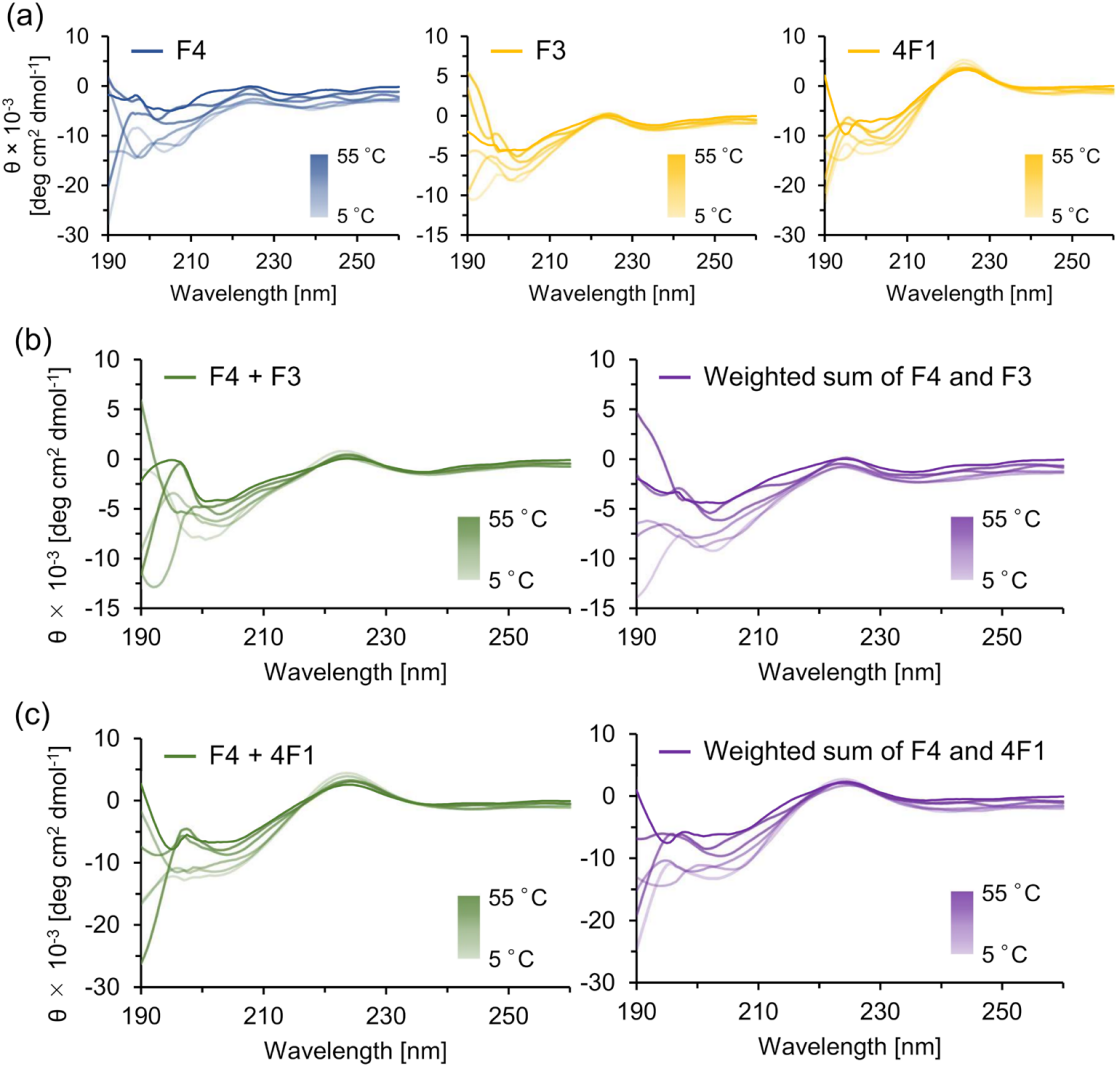
CD spectra upon heating. (a) Spectra of single‐component F4 (0.0210 mg/mL, left), F3 (0.0790 mg/mL, middle), and 4F1 (0.0508 mg/mL, right), (b) Mixture of F4 + F3 (F4 0.0210 mg/mL and F3 0.0790 mg/mL, left) and the weighted sum of their individual spectra shown in panel a (right), and (c) Mixture of F4 + 4F1 (F4 0.0210 mg/mL and 4F1 0.0508 mg/mL, left) and the weighted sum of their individual spectra shown in panel a (right).

The single‐component F4, F3, and 4F1 showed CD spectral features similar to those obtained for H‐(FPGVG)_n_‐NH_2_ and [α‐E(F1)]_n_‐F1 in previous studies (Figure 6a). Among them, F4 showed somewhat less distinct CD spectra than the other peptides, likely because of the lower concentration used in this analysis (0.0210 mg/mL). Nonetheless, each peptide showed a decrease in the intensity of the negative peak and an increase in the shoulder peak at approximately 210 nm upon heating, indicating structural changes from PPII to β‐structures (sheet and turn) as the temperature increased.

The F4 + F3 and F4 + 4F1 binary mixtures exhibited CD bands similar to those observed for short ELPs in previous studies (Figure 6b,c, left). Their CD spectra were closely reproduced by the theoretical spectra (*θ* in deg cm^2^ dmol^−1^ and CD in mdeg) calculated from the corresponding single‐component data of F4 and the partner (F3 or 4F1) using Equation (1) (Figure 6b,c, right). To enable a quantitative comparison, the root mean square deviation (RMSD) values were calculated over a range of 195–260 nm. The RMSD values were approximately 620–1500 deg cm^2^ dmol^−1^ (0.67–1.62 mdeg) for F4 + F3 and approximately 220–1320 deg cm^2^ dmol^−1^ (0.24–2.75 mdeg) for F4 + 4F1, respectively (Table S3). These RMSD values supported the high similarity between the theoretical and experimental spectra of the two‐component samples [46].

The spectral similarity between the measured binary mixtures and the theoretical spectra calculated from the individual components indicates that, under the conditions used for CD measurement, mixing induced minimal structural changes or heterotypic intermolecular interactions and did not significantly alter the respective secondary structures of the peptides [39]. However, the shift in the temperature at which turbidity increased (as observed by turbidimetry), the differing depletion trends in UPLC‐MS analysis, and the disparity in the number and density of aggregates observed using microscopy showed that the phase separation of the two components in the mixture was not entirely independent. Therefore, these observations indicate that the intermolecular interactions between the two components are influenced by the peptide concentration and ionic strength, which can modulate the stepwise nature of their phase behavior. The importance of concentration is also supported by a previous study by Ghoorchian and Holland, which reported that mixtures of (GVGVP)_40_ and trimeric (GVGVP)_40_ exhibited two sequential transitions at low concentration ranges [47]. These results highlight the importance of the component composition, concentration, and solution conditions in designing the phase behavior of multicomponent peptide systems.

In addition, CD spectra were obtained for each sample during cooling. The results revealed structural reversibility upon temperature cycling (Figures S13 and S14, Table S3).

**FIGURE 7:**
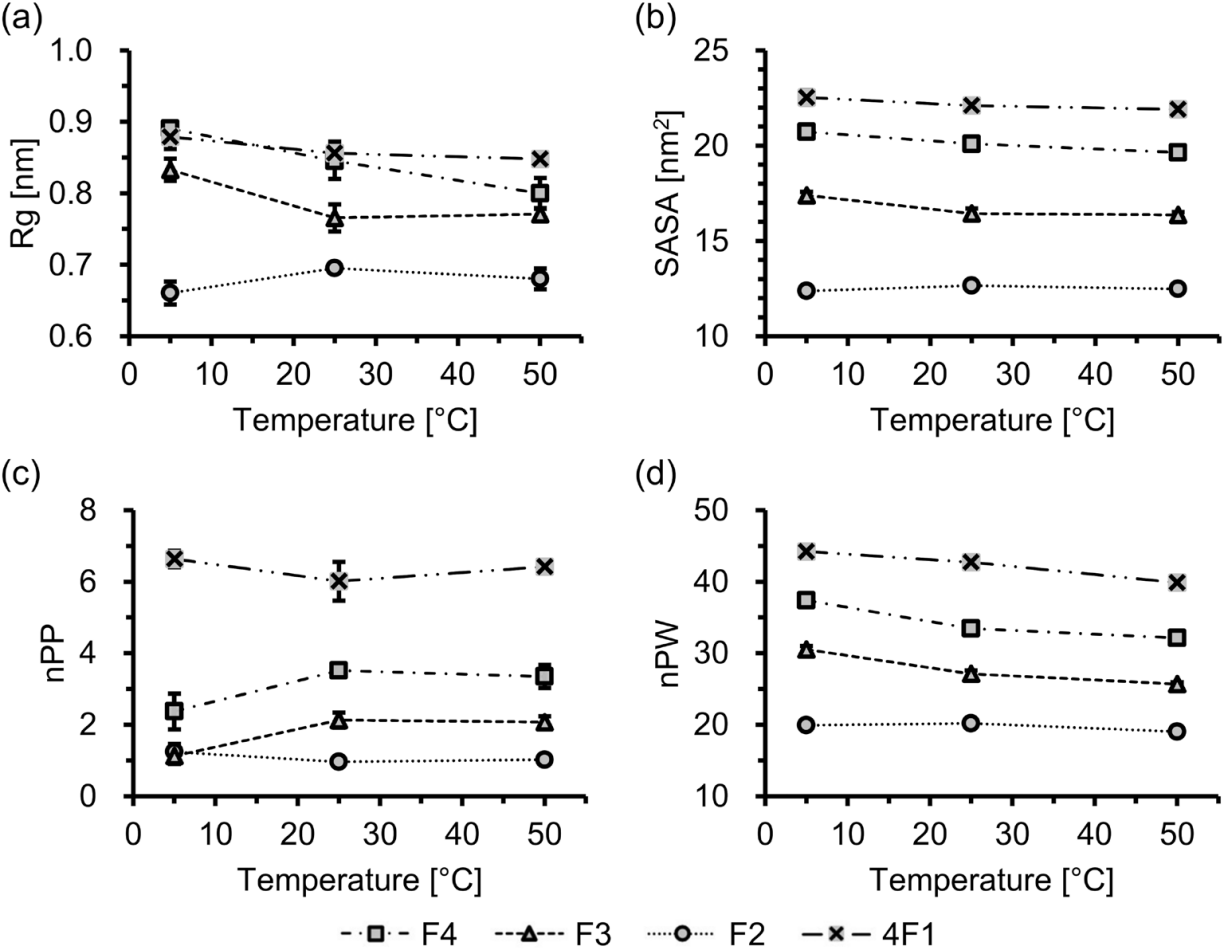
Temperature‐dependent structural parameters of single ELP molecules. (a) Radius of gyration (Rg), (b) solvent‐accessible surface area (SASA), (c) number of intramolecular hydrogen bonds (nPP), and (d) number of hydrogen bonds between peptide and water molecules (nPW) for F4 (square), F3 (triangle), F2 (circle), and 4F1 (cross) in the absence of NaCl. Data represent the average of three independent simulation runs, with standard errors (SE) shown as error bars. Values obtained at 5°C, 25°C, and 50°C are connected by lines for visual clarity.

To obtain insight into the mechanism underlying the stepwise transition, MD simulations were performed without and with 3 M NaCl (Figure 7 and Figure S15). Under these salt conditions, similar trends were observed in the parameters of radius of gyration (Rg), solvent‐accessible surface area (SASA), number of intramolecular hydrogen bonds (nPP), and number of hydrogen bonds between peptide and water molecules (nPW) with temperature changes. The MD data revealed distinct temperature‐dependent conformational changes in F4, F3, F2, and 4F1. Single molecules F4, F3, and 4F1 exhibited decreasing trends in Rg and SASA upon heating, indicating that each ELP monomer became more compact at elevated temperatures (Figure 7a,b). In contrast, both Rg and SASA of F2 increased upon heating from 5°C to 25°C, revealing that F2 adopts a more extended conformation under these conditions (Figure 7a,b). nPP increased, and nPW decreased with increasing temperature for F4 and F3 (Figure 7c,d), indicating that these ELPs underwent dehydration at high temperatures. Similarly, nPW of 4F1 also decreased upon heating (Figure 7d), consistent with the behavior of F4 and F3; however, its nPP remained relatively unchanged at 5°C–50°C (Figure 7c). In contrast, F2 exhibited subtle changes in nPP and nPW in this temperature range (Figure 7c,d). Overall, F2 is inherently compact and exhibits only subtle conformational changes, making F2 less likely to undergo temperature‐induced dehydration or form intramolecular hydrogen bonds in a single molecule.

These findings indicate that ELPs exhibit different characteristics in response to changes in temperature. Subtle differences in chain length and topology shift the balance between intra‐ and intermolecular interactions, resulting in partially independent phase transitions. These differences could also contribute to the observed trends in scattering intensity in the DLS measurements.

## 4 Conclusion

Understanding the phase behavior of multicomponent systems comprised of short‐chain LLPS‐capable peptides is crucial for the rational design of functional peptide‐based materials. This study investigated the phase behavior of mixtures of synthetic short‐chain ELPs that differed either by only one or two pentapeptide repeat units in chain length or by topology, while maintaining the same number of repeats. Both linear ELPs with Δ*n* = 1 or 2 and branched ELPs showed stepwise phase separation, similar to that previously observed in long‐chain ELPs with notable differences in chain length or sequence. In addition, although the mixtures exhibited stepwise phase separation, our results revealed that heterotypic inter‐peptide interactions affect the phase separation properties.

Under temperature gradients or switches, these stepwise transitions enable sequential separation and spatiotemporal organization of ELP‐fused components and coexisting molecules within a mixture. Therefore, this study deepens the understanding of phase behavior in mixed ELP systems and facilitates the use of multicomponent systems based on synthetic short‐chain intrinsically disordered protein mimics.

## Author Contributions

**Naoki Tanaka:** conceptualization, methodology, investigation, formal analysis, writing – original draft, writing – review and editing, funding acquisition. **Keitaro Suyama:** writing‐review and editing, co‐supervision. **Elissa Mai:** investigation, writing – review and editing. **Takeru Nose:** conceptualization, writing – review and editing, supervision, funding acquisition.

## Funding

This work was supported by Japan Society for the Promotion of Science, JP23KJ1744, JP24K03120.

## Conflicts of Interest

The authors declare no conflicts of interest.

## Supporting information


**Table S1:** Yield, retention time, and *m*/*z* of ELPs.
**Table S2:** *T*_t_ values of single‐component ELPs.
**Table S3:** RMSD of measured and calculated CD spectra.
**Figure S1:** UPLC‐MS chromatograms.
**Figure S2:** Turbidity profiles of single‐component ELPs.
**Figure S3:** Peptide concentration dependency of single‐component ELPs.
**Figure S4:** Pictures of ELP mixtures after incubation and after centrifugation.
**Figure S5:** Microscopy images of F4 + F3.
**Figure S6:** Microscopy images of F3 + F2.
**Figure S7:** Microscopy images of F4 + 4F1.
**Figure S8:** Microscopy images of mixtures in phosphate buffer with 1 M NaCl.
**Figure S9:** DLS results in number.
**Figure S10:** Scattering intensity of single‐component and mixture samples.
**Figure S11:** DLS autocorrelation curves.
**Figure S12:** CD spectra upon heating shown in mdeg.
**Figure S13:** CD spectra upon cooling shown in mdeg.
**Figure S14:** CD spectra upon cooling.
**Figure S15:** MD simulation under 3 M NaCl.

## Data Availability

The data that support the findings of this study are available in the Supporting Information of this article. Additional data related to this study are available from the corresponding author upon reasonable request.
