# Supplementary material for: Stepwise LCST‐Type Phase Separation in Mixtures of Short‐Chain Elastin‐Like Peptides With Minimal Structural Differences
Source: Biopolymers. 2026 Feb 13;117(2):e70084. doi: 10.1002/bip.70084 (PMC12903191; doi:10.1002/bip.70084)
Supplement: Supplementary file 1 — Table S1: Yield, retention time, and m/z of ELPs. Table S2: T t values of single‐component ELPs. Table S3: RMSD of measured and calculated CD spectra. Figure S1: UPLC‐MS chromatograms. Figure S2: Turbidity profiles of single‐component ELPs. Figure S3: Peptide concentration dependency of single‐component ELPs. Figure S4: Pictures of ELP mixtures after incubation and after centrifugation. Figure S5: Microscopy images of F4 + F3. Figure S6: Microscopy images of F3 + F2. Figure S7: Microscopy images of F4 + 4F1. Figure S8: Microscopy images of mixtures in phosphate buffer with 1 M NaCl. Figure S9: DLS results in number. Figure S10: Scattering intensity of single‐component and mixture samples. Figure S11: DLS autocorrelation curves. Figure S12: CD spectra upon heating shown in mdeg. Figure S13: CD spectra upon cooling shown in mdeg. Figure S14: CD spectra upon cooling. Figure S15: MD simulation under 3 M NaCl. [file BIP-117-e70084-s001.docx]

**Supporting Information**

**Stepwise LCST-Type Phase Separation in Mixtures of Short-Chain Elastin-Like Peptides with Minimal Structural Differences**

Naoki Tanaka,^1^ Keitaro Suyama,^1,2^ Elissa Mai,^1^ and Takeru Nose^1,2*^

^1^ Department of Chemistry, Faculty and Graduate School of Science, Kyushu University, Fukuoka, 819-0395, Japan.

^2^ Faculty of Arts and Science, Kyushu University, Fukuoka, 819-0395, Japan.

Manuscript Correspondence:

Prof. Takeru Nose

Tel: +81-92-802-6025

Fax: +81-92-802-6025

e-mail: nose@artsci.kyushu-u.ac.jp

**Supporting Method**

Molecular dynamics (MD) simulation2

Supporting Tables and Figures

Table S1. Yield, retention time, and *m/z* of ELPs3

Table S2. *T*_t_ values of single-component ELPs4

Table S3. RMSD of measured and calculated CD spectra5

Figure S1. UPLC-MS chromatograms6

Figure S2. Turbidity profiles of single-component ELPs7

Figure S3. Peptide concentration dependency of single-component ELPs8

Figure S4. Pictures of ELP mixtures after incubation and after centrifugation9

Figure S5. Microscopy images of F4 + F310

Figure S6. Microscopy images of F3 + F211

Figure S7. Microscopy images of F4 + 4F112

Figure S8. Microscopy images of mixtures in phosphate buffer with 1 M NaCl13

Figure S9. DLS results in number14

Figure S10. Scattering intensity of single-component and mixture samples15

Figure S11. DLS autocorrelation curves16

Figure S12. CD spectra upon heating shown in mdeg17

Figure S13. CD spectra upon cooling shown in mdeg18

Figure S14. CD spectra upon cooling19

Figure S15. MD simulation under 3 M NaCl20

**SUPPORTING METHODS**

**Molecular Dynamics (MD) simulation.** MD simulations of single molecules were performed for each ELPs using a DELL PRECISION T3610 workstation (Dell Inc. Round Rock, TX, USA). The simulations were performed in GROMACS 2019 with AMBER99SB-ILDN force field and TIP3P explicit solvent model. Initial conformations (.pdb file) were generated by Discovery Studio 4.0 software (Dassault Systèmes BIOVIA, San Diego, CA, USA) and translated into .gro file by gmx pdb2gmx command in GROMACS. The model of ELP was placed in a cubic box (F4 in 6 × 6 × 6 nm^3^, F3 and [α-E(F1)]_3_-F1 in 5 × 5 × 5 nm^3^, and F2 in 4 × 4 × 4 nm^3^) and solvated with explicit TIP3P water molecules by gmx solvate. For simulations in the absence of NaCl, appropriate numbers of water molecules were replaced with a Cl^-^ via gmx genion to neutralize the total charge of the system. For simulations in the presence of 3 M NaCl, the minimum distance parameter (-rmin) was set to 0.51 nm (default 0.6 nm) to enable successful insertion of Na^+^ and Cl^-^ without exhausting solvent replacement sites. The simulations were performed at 278, 298, and 323 K (5, 25, and 50 °C). These systems contained two minimization steps, a heating step, an equilibrium step, and a production step. The first minimization was performed by steepest descent algorithm, using a maximum of 10,000 steps, the maximum step size (emstep) of 0.01 nm, the tolerance (emtol) of 10.0 kJ mol^-1^ nm^-1^, and no constraints; The second minimization was performed by conjugate gradient algorithm, using a maximum of 20,000 steps, emstep of 0.01 nm, emtol of 10.0 kJ mol^-1^ nm^-1^, and no constraints; heating step: parameters are 500,000 steps, time step 2 fs, initial temperature 0 K, target temperature (278/298/323 K), LINCS constraint on h-bond atoms, annealing type single, annealing npoints 2, and annealing time 200 ps; an equilibration step: parameters are 500,000 steps with a time step of 2 fs, target temperature (278/298/323 K), LINCS constraint to h-bond atoms, and reference pressure 1.0 bar; and finally, a production step consisting of 25,000,000 steps (50 ns) for F4, F3, and [α-E(F1)]_3_-F1, and 50,000,000 steps (100 ns) for F2, time step 2 fs, target temperature (278/298/323 K), pressure coupling decay time (tau-p) = 1.0, LINCS constraint to h-bond atoms, nonbond list radius (rcoulomb, rvdw) = 1.0 nm, nonbond lower cutoff distance (rlist) = 1.0 nm, electrostatics = particle-mesh Ewald (PME) method, dynamics integrator = leapfrog, and random number seed = 1732. Trajectories (50,000 frames for F4, F3, and [α-E(F1)]_3_-F1, and 100,000 frames for F2) were processed by gmx trjconv command to correct the break in the molecular structure due to periodic boundary condition. During this process, the number of frames were reduced to 1/10 (5,000 or 10,000 frames). Then, the radius of gyration (Rg), solvent accessible surface areas (SASA), the number of intramolecular hydrogen bonds (nPP), and the number of hydrogen bonds between water and peptide (nPW) were analyzed using the processed trajectory files with omitting the first 10 ns.

**Reference**

[1] B. Hess, C. Kutzner, D. van der Spoel, E. Lindahl, *J. Chem. Theory Comput.* **2008**, *4*, 435.

**SUPPORTING TABLES AND FIGURES**

Table S1. Yield, retention time, and *m/z* of ELPs.

| ELPs | Yields [%] | *R*_t_ [min]^a^ | MS (ESI) *m/z*  (calcd/found)^b^ |
| --- | --- | --- | --- |
| F4 | 75 | 1.982^c^ | 924.59 [M + 2H]^2+^/924.48 |
| F3 | 71 | 1.379^c^ | 695.82 [M + 2H]^2+^/695.83 |
| F2 | 70 | 2.376^d^ | 933.10 [M + H]^+^/932.60 |
| [α-E(F1)]_3_-F1 (4F1) | 61 | 1.812^c^ | 1116.78 [M + 2H]^2+^/1116.68 |

^a^ Retention times in UPLC. The solvent system for UPLC consisted of 0.1% formic acid aqueous solution (v/v, solvent A) and 0.1% formic acid in acetonitrile (v/v, solvent B).

^b^ *m/z* in UPLC-MS. The calculated values are for the average mass.

^c^ The peptides were monitored in a linear gradient of solvent B in solvent A (24% to 56%) over 4.23 min.

^d^ The peptide was monitored in a linear gradient of solvent B in solvent A (10% to 50%) over 4.51 min.

Table S2. *T*_t_ values of single-component ELPs.

| ELPs | ELP concentration [mM] | NaCl concentration [M] | *T*_t_ [°C] |
| --- | --- | --- | --- |
| F4 | 0.2 | 3 | 17.56 ± 0.49 |
|  | 3 | 1 | 20.02 ± 0.19 |
|  | 2.5 | 1 | 22.88 ± 0.43 |
|  | 1 | 1 | 44.66 ± 0.30 |
| F3 | 2 | 3 | 21.09 ± 1.32 |
|  | 1.5 | 3 | 26.57 ± 1.44 |
|  | 1 | 3 | 39.66 ± 1.09 |
|  | 15 | 1 | 24.78 ± 1.68 |
|  | 10 | 1 | 36.15 ± 1.03 |
|  | 7.5 | 1 | 44.98 ± 0.88 |
| 4F1 | 1 | 3 | 11.61 ± 0.17 |
|  | 0.5 | 3 | 26.28 ± 0.21 |
|  | 0.4 | 3 | 33.67 ± 0.53 |
|  | 10 | 1 | 17.56 ± 0.60 |
|  | 7.5 | 1 | 33.27 ± 0.64 |
|  | 5 | 1 | 25.25 ± 0.89 |
| F2 | 30 | 3 | 17.02 ± 1.18 |
|  | 20 | 3 | 34.03 ± 1.09 |
|  | 15 | 3 | 50.35 ± 2.00 |

Table S3. RMSD of measured and calculated CD spectra.

|  |  | F4 + F3 | | F4 + 4F1 | |
| --- | --- | --- | --- | --- | --- |
|  |  | Heating | Cooling | Heating | Cooling |
| 5 °C | deg cm^2^ dmol^-1^ | 1322.47 | 2060.27 | 1245.02 | 2132.73 |
|  | mdeg | 1.43 | 2.23 | 1.29 | 2.62 |
| 15 °C | deg cm^2^ dmol^-1^ | 1499.54 | 2368.46 | 1318.13 | 2640.26 |
|  | mdeg | 1.62 | 2.56 | 1.05 | 2.26 |
| 25 °C | deg cm^2^ dmol^-1^ | 1067.39 | 1921.41 | 976.06 | 2579.41 |
|  | mdeg | 1.15 | 2.08 | 1.64 | 1.98 |
| 35 °C | deg cm^2^ dmol^-1^ | 836.39 | 1491.44 | 798.85 | 1530.74 |
|  | mdeg | 0.90 | 1.61 | 1.34 | 1.17 |
| 45 °C | deg cm^2^ dmol^-1^ | 624.24 | 890.91 | 668.39 | 956.18 |
|  | mdeg | 0.67 | 0.96 | 2.75 | 0.92 |
| 55 °C | deg cm^2^ dmol^-1^ | 697.74 |  | 222.83 |  |
|  | mdeg | 0.75 |  | 0.24 |  |

RMSD were calculated in the range of 195–260 nm.


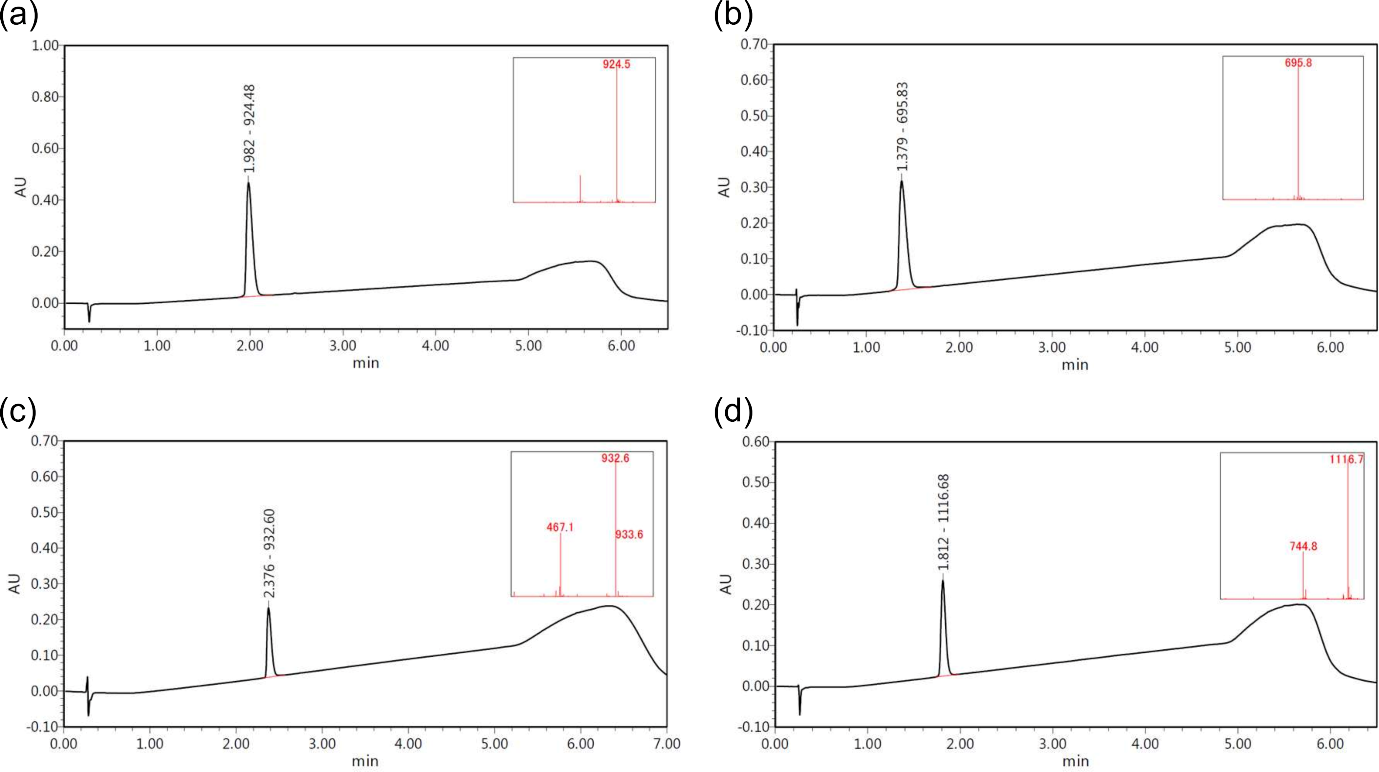


Figure S1. UPLC-MS chromatograms.

(a) F4, (b) F3, (c) F2, and (d) 4F1. The subscripts for each peak indicate the *R*_t_ and the *m/z* detected. The inset panels show MS spectra of the corresponding peak determined by ACQUITY QDa mass spectrometer. The solvent system for UPLC consisted of 0.1% formic acid aqueous solution (v/v, solvent A) and 0.1% formic acid in acetonitrile (v/v, solvent B). F4, F3, and 4F1 were monitored in a linear gradient of solvent B in solvent A (24% to 56%) over 4.23 min. F2 was monitored in a linear gradient of solvent B in solvent A (10% to 50%) over 4.51 min.


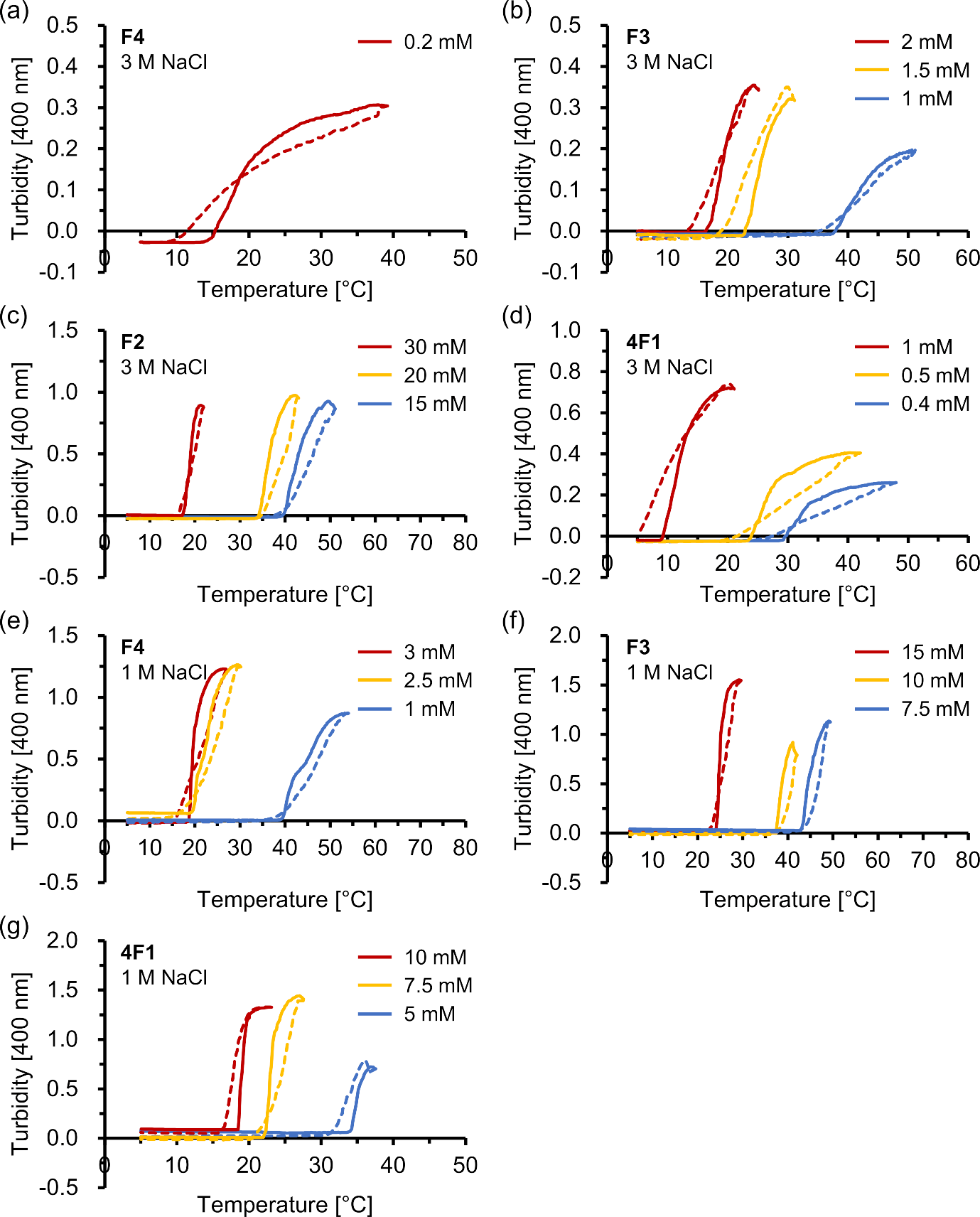


Figure S2. Turbidity profiles of single-component ELPs.

Turbidity profiles of F4 (panels a, e), F3 (b, f), F2 (c), and 4F1 (d, g). In panels (a), (b), (c), and (d), peptides were dissolved in phosphate buffer with 3 M NaCl. In (e), (f), and (g), peptides were dissolved in phosphate buffer with 1 M NaCl.


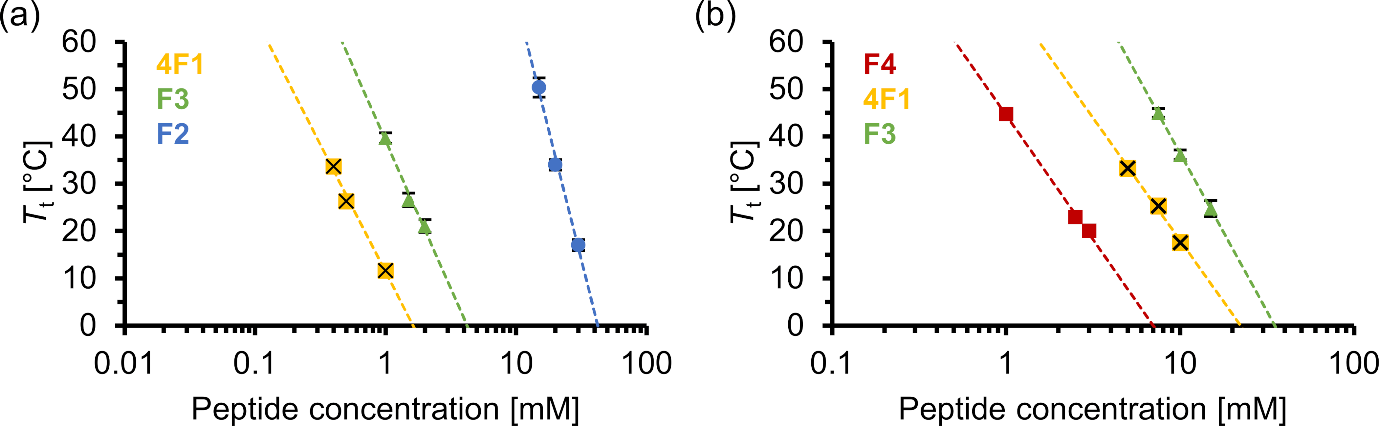


Figure S3. Peptide concentration dependency of single-component ELPs.

Peptides were dissolved in phosphate buffer with 3 M NaCl in (a) and 1 M NaCl in (b). Dotted lines represent the linear fit to the experimental *T*_t_ values, following the equation *T*_t_ = *a* log(*C*_ELP_) + *b*, where *C*_ELP_ is the ELP concentration, and the *a* and *b* are constant values.


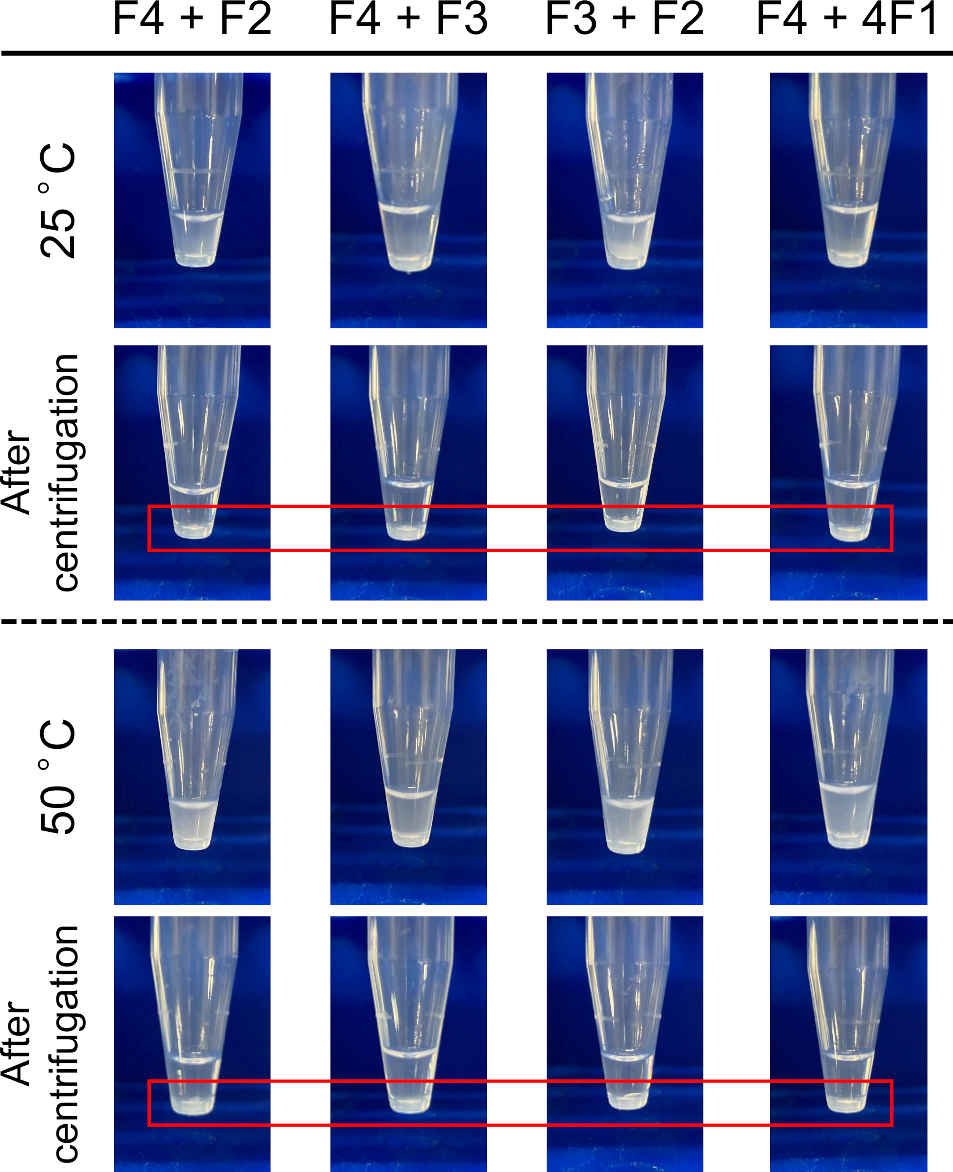


Figure S4. Pictures of ELP mixtures after incubation and after centrifugation.

The peptides were dissolved in phosphate buffer with 3 M NaCl as follows: F4 + F2 (0.2 mM F4 and 15 mM F2), F4 + F3 (0.2 mM F4 and 1 mM F3), F3 + F2 (2 mM F3 and 15 mM F2), and F4 + 4F1 (0.2 mM F4 and 0.4 mM 4F1. Images taken immediately after incubation at 25 °C or 50 °C, and after centrifugation, are shown. The red box highlights small volumes of centrifuged peptides, which were difficult to collect and therefore challenging to analyze by UPLC-MS.


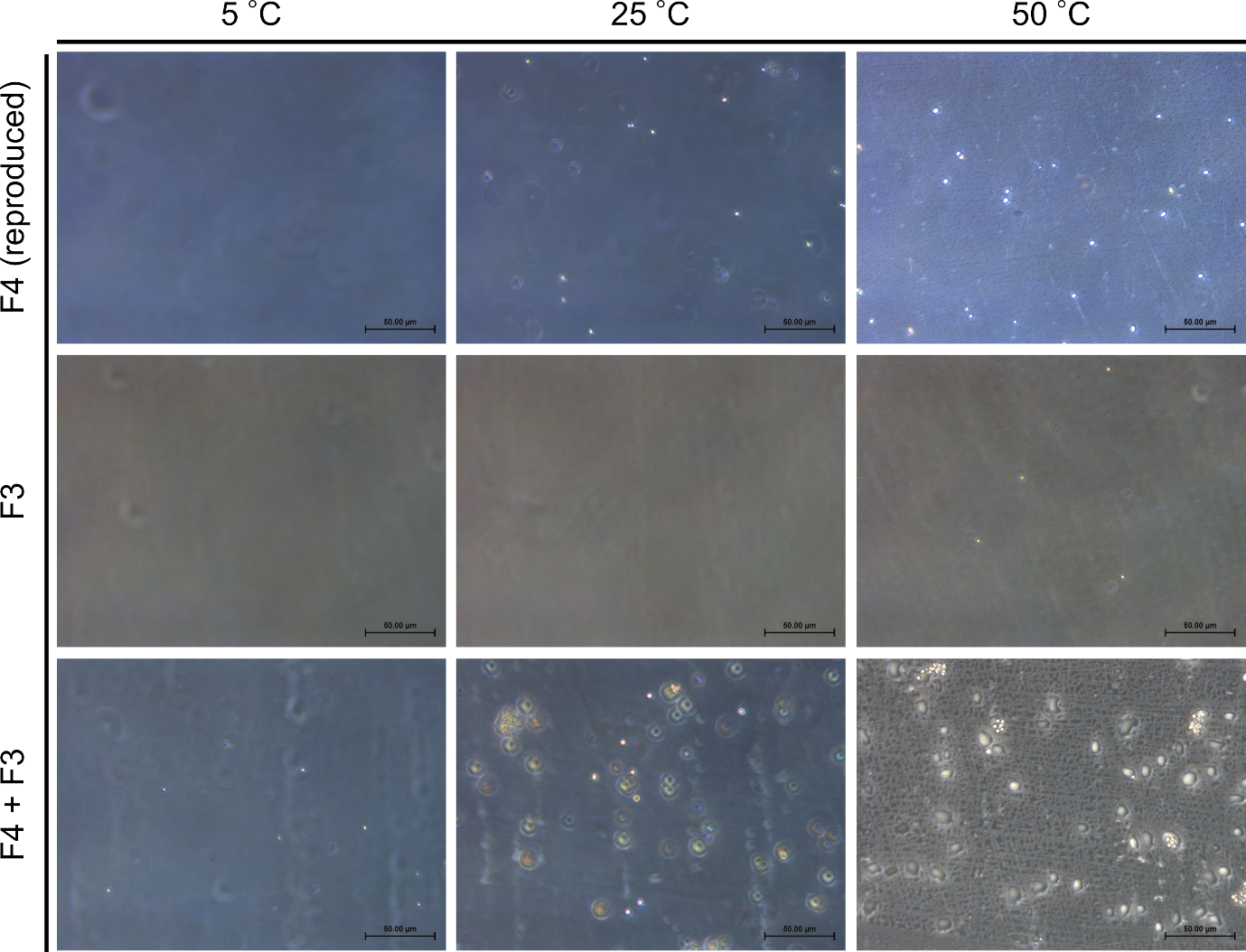
Figure S5. Microscopy images of F4 + F3.

For single-component samples, F4 and F3 were dissolved at 0.2 mM and 1 mM, respectively. In the mixed sample (F4 + F3), F4 and F3 were dissolved at 0.2 mM and 1 mM, respectively. Samples were prepared in phosphate buffer supplemented with 3 M NaCl. Pictures were taken after 2 min of equilibration at target temperatures. Scale bars indicate 50 μm. The images of F4 (top row) are the same as those in Figure 4 and are reproduced for ease of comparison.


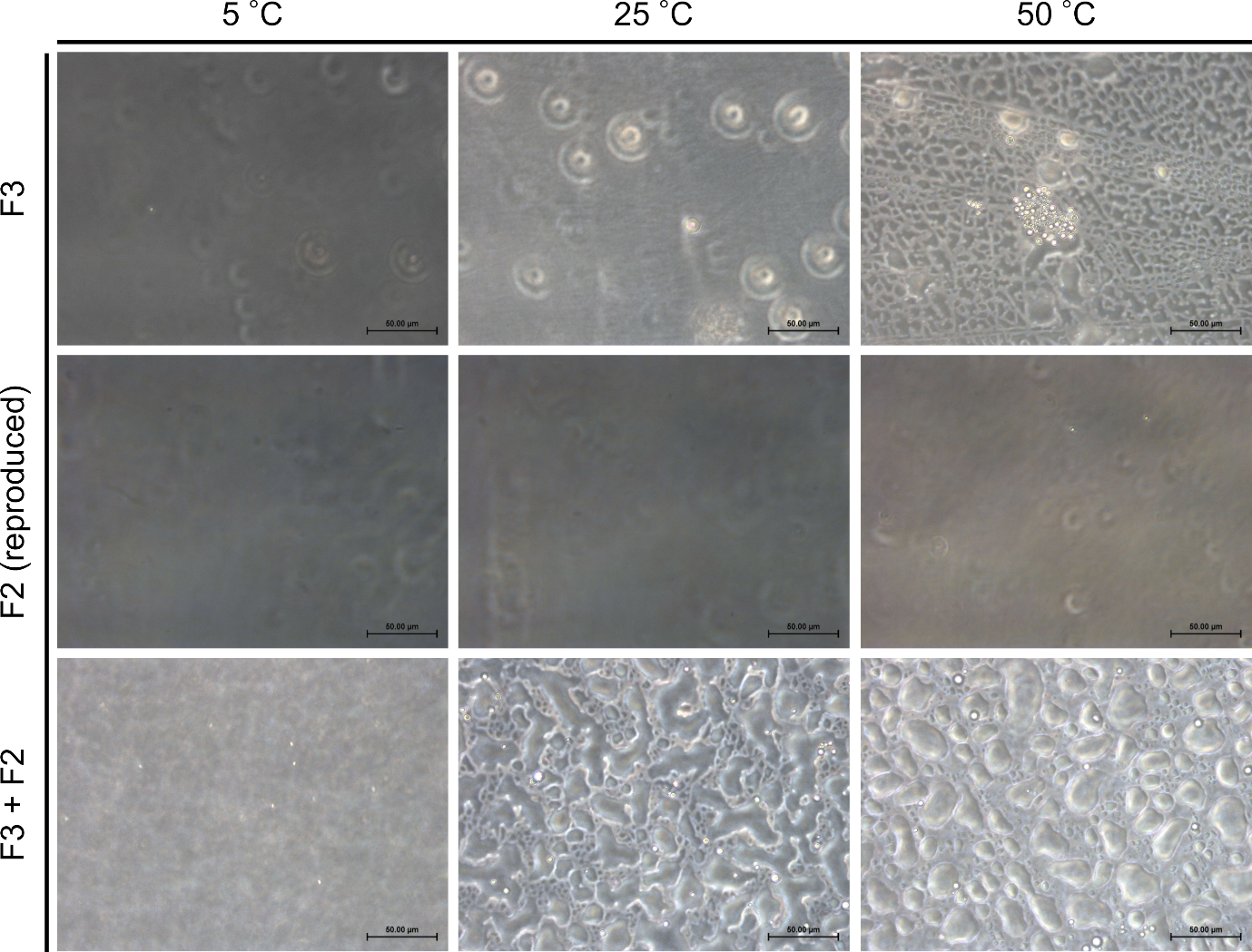
Figure S6. Microscopy images of F3 + F2.

For single-component samples, F3 and F2 were dissolved at 2 mM and 15 mM, respectively. In the mixed sample (F3 + F2), F3 and F2 were dissolved at 2 mM and 15 mM, respectively. Samples were prepared in phosphate buffer supplemented with 3 M NaCl. Pictures were taken after 2 min of equilibration at target temperatures. Scale bars indicate 50 μm. The images of F2 (middle row) are the same as those in Figure 4 and are reproduced for ease of comparison.


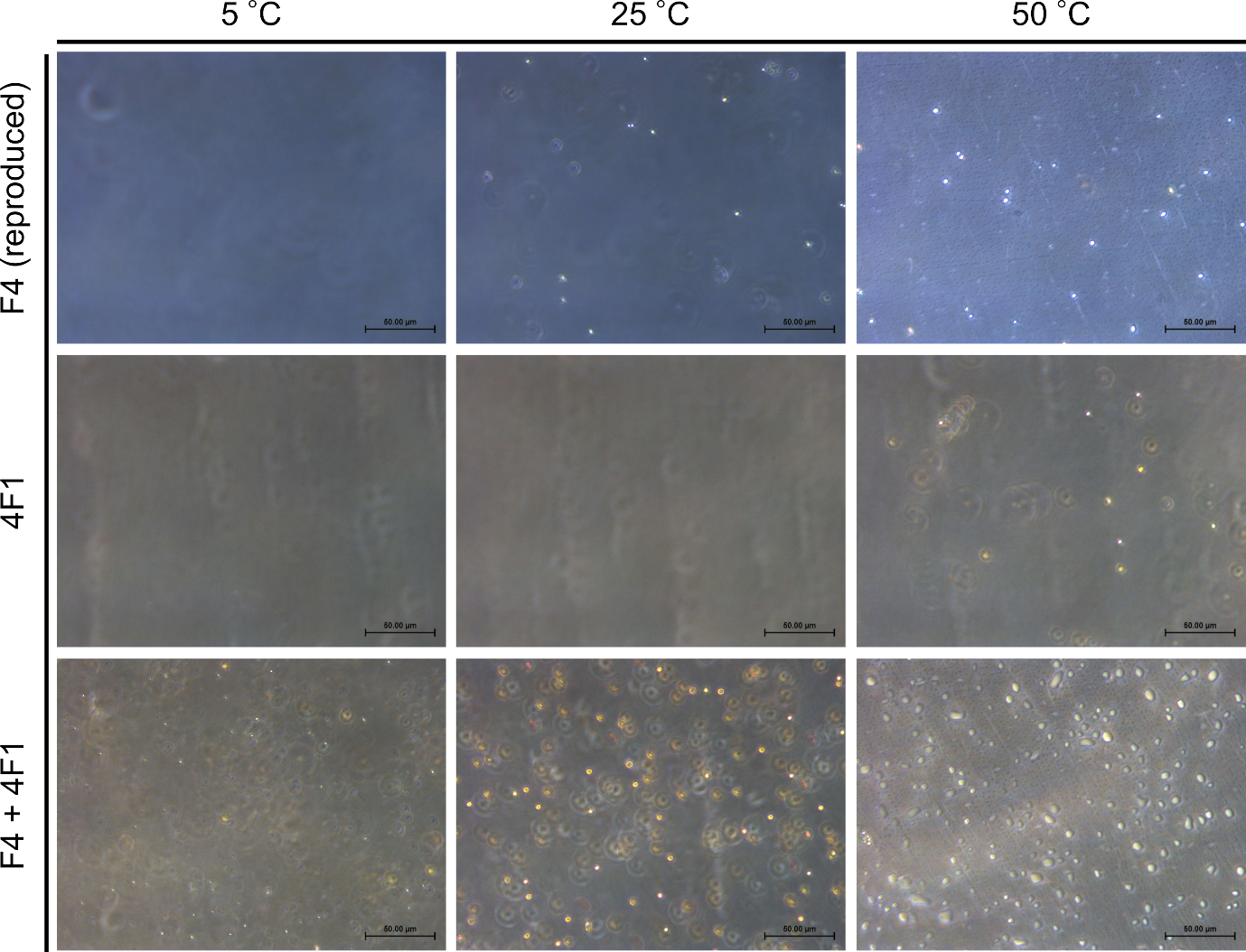
Figure S7. Microscopy images of F4 + 4F1.

For single-component samples, F4 and 4F1 were dissolved at 0.2 mM and 0.4 mM, respectively. In the mixed sample (F4 + 4F1), F4 and 4F1 were dissolved at 0.2 mM and 0.4 mM, respectively. Samples were prepared in phosphate buffer supplemented with 3 M NaCl. Pictures were taken after 2 min of equilibration at target temperatures. Scale bars indicate 50 μm. The images of F4 (top row) are the same as those in Figure 4 and are reproduced for ease of comparison.


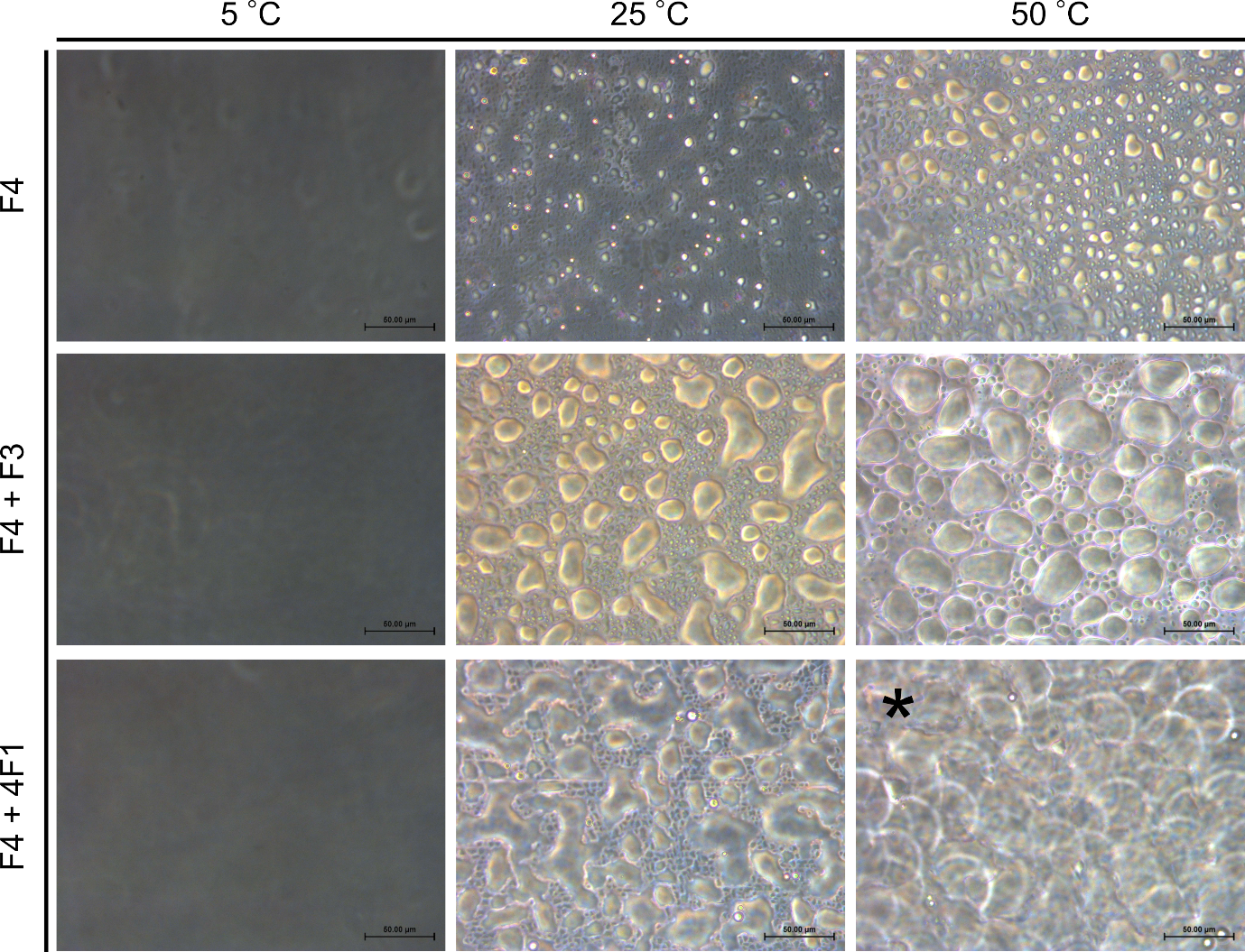


Figure S8. Microscopy images of mixtures in phosphate buffer with 1 M NaCl.

Samples were prepared in phosphate buffer supplemented with 1 M NaCl. The single-component F4 was dissolved at 3 mM. In F4 + F3, F4 and F3 were dissolved at 3 mM and 10 mM, respectively. In F4 + 4F1, F4 and 4F1 were dissolved at 3 mM and 5 mM, respectively. A clear, focused image could not be obtained for F4 + 4F1 at 50 °C (*) because of the dense and large number of precipitates. Pictures were taken after 2 min of equilibration at target temperatures. Scale bars indicate 50 μm.


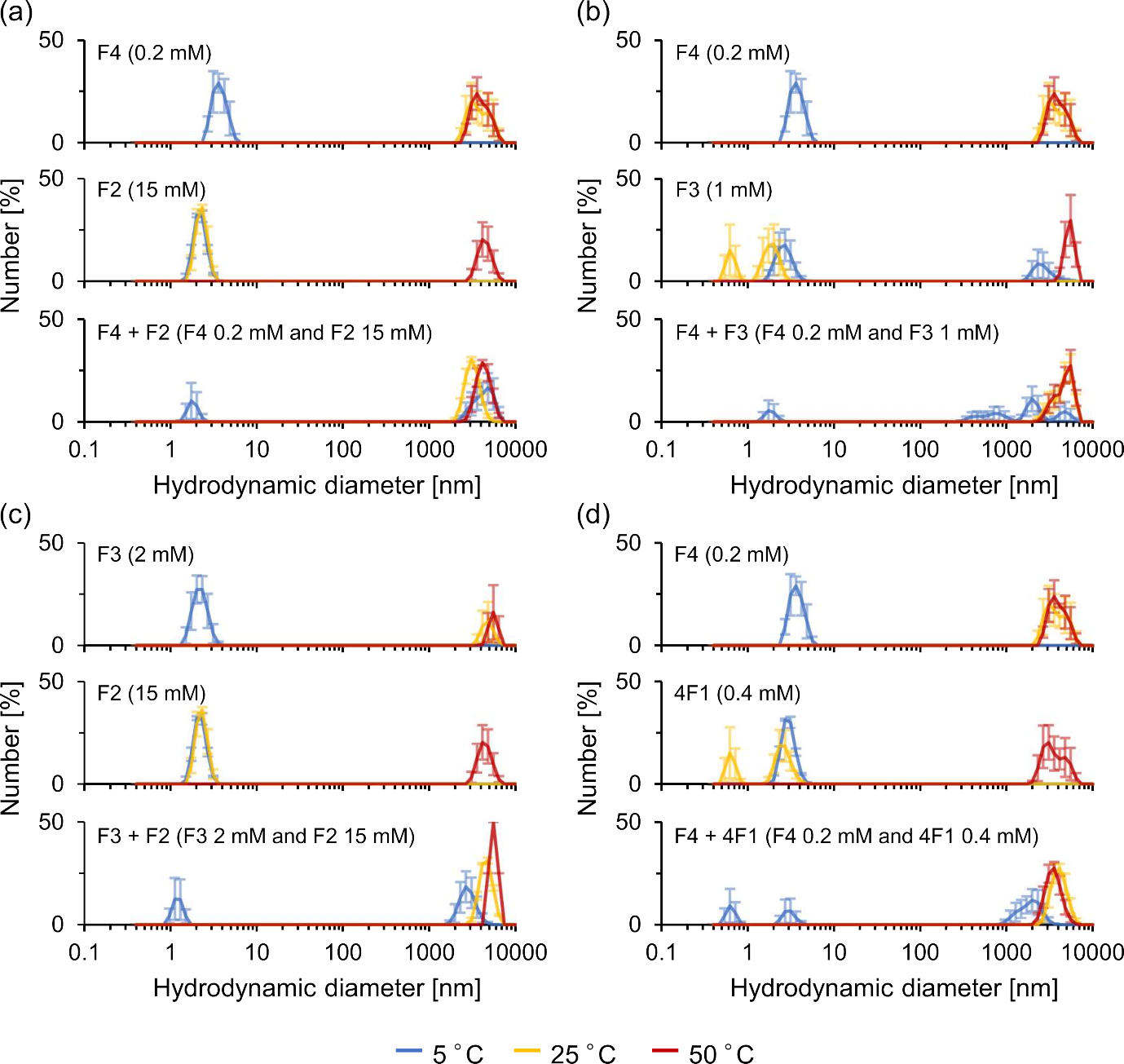


Figure S9. DLS results in number.

(a) F4, F2, and F4 + F2, (b) F4, F3, and F4 + F3, (c) F3, F2, and F3 + F2, and (d) F4, 4F1, and F4 + 4F1. Samples were prepared in phosphate buffer supplemented with 3 M NaCl. Results are shown in number (%) with standard errors.


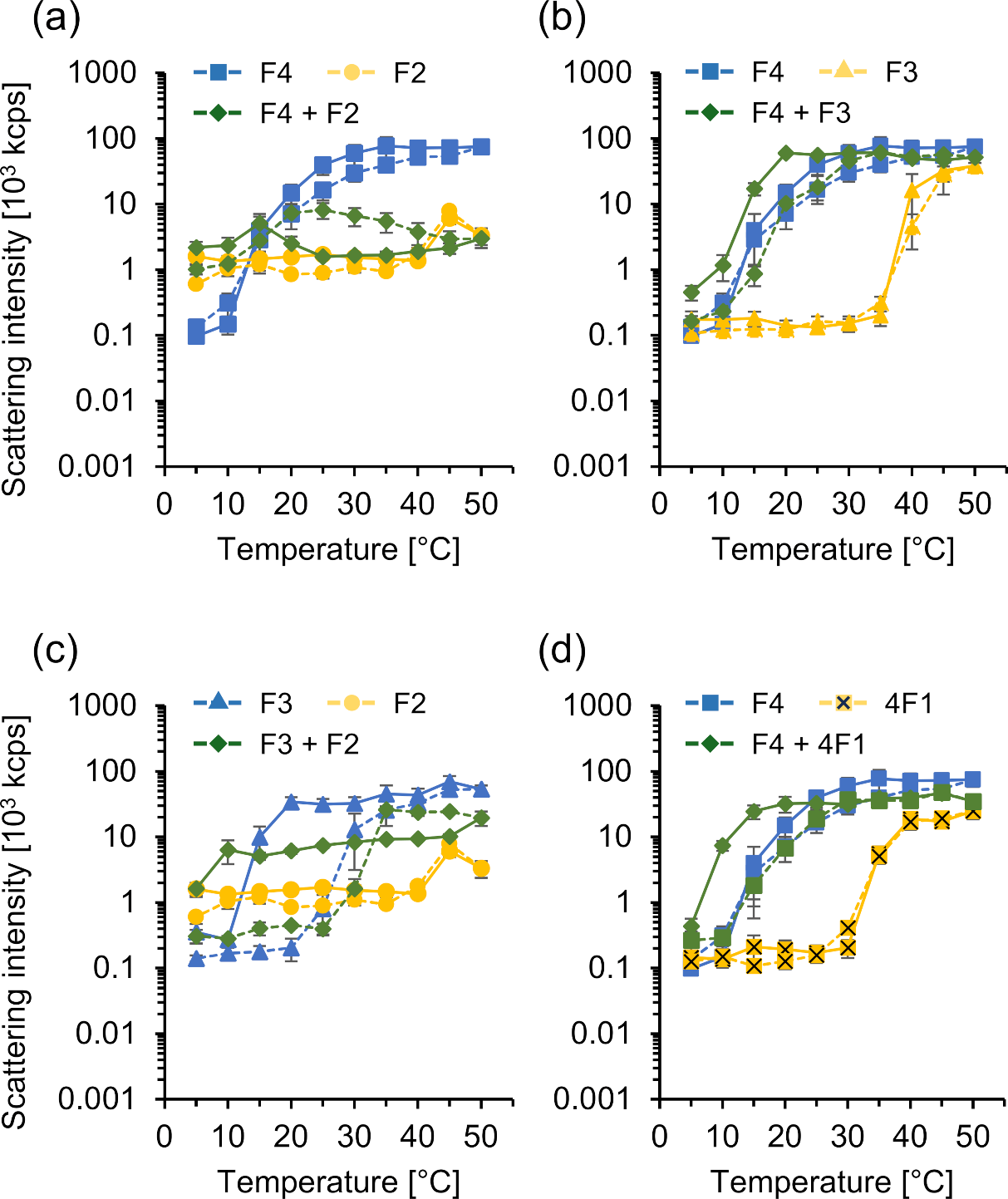


Figure S10. Scattering intensity of single-component and mixture samples.

(a) F4 0.2 mM, F2 15 mM, and F4 + F2 (F4 0.2 mM and F2 15 mM), (b) F4 0.2 mM, F3 1 mM, F4 + F3 (F4 0.2 mM and F3 1 mM), (c) F3 2 mM, F2 15 mM, and F3 + F2 (F3 2 mM and F2 15 mM), (d) F4 0.2 mM, 4F1 0.4 mM, F4 + 4F1 (F4 0.2 mM and 4F1 0.4 mM). Samples were prepared in phosphate buffer supplemented with 3 M NaCl. Solid lines and dashed lines represent heating and cooling, respectively.


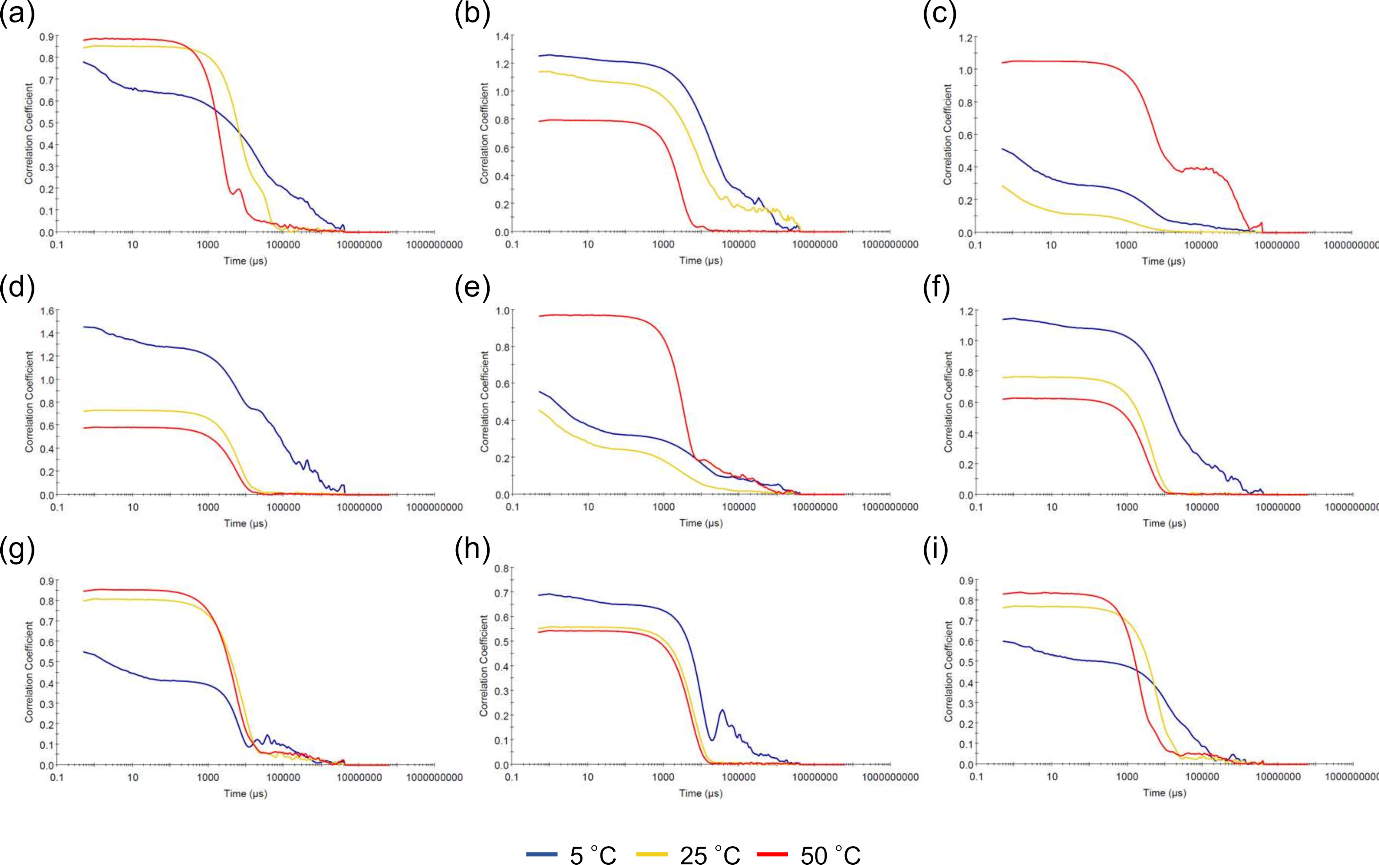


Figure S11. DLS autocorrelation curves.

(a) F4 0.2 mM, (b) F2 15 mM, (c) F3 1 mM, (d) F3 2 mM, (e) 4F1 0.4 mM, (f) F4 + F2 (F4 0.2 mM and F2 15 mM), (g) F4 + F3 (F4 0.2 mM and F3 1 mM), (h) F3 + F2 (F3 2 mM and F2 15 mM), (i) F4 +4F1 (F4 0.2 mM and 4F1 0.4 mM). Peptides were dissolved in filtered phosphate buffer supplemented with 3 M NaCl.


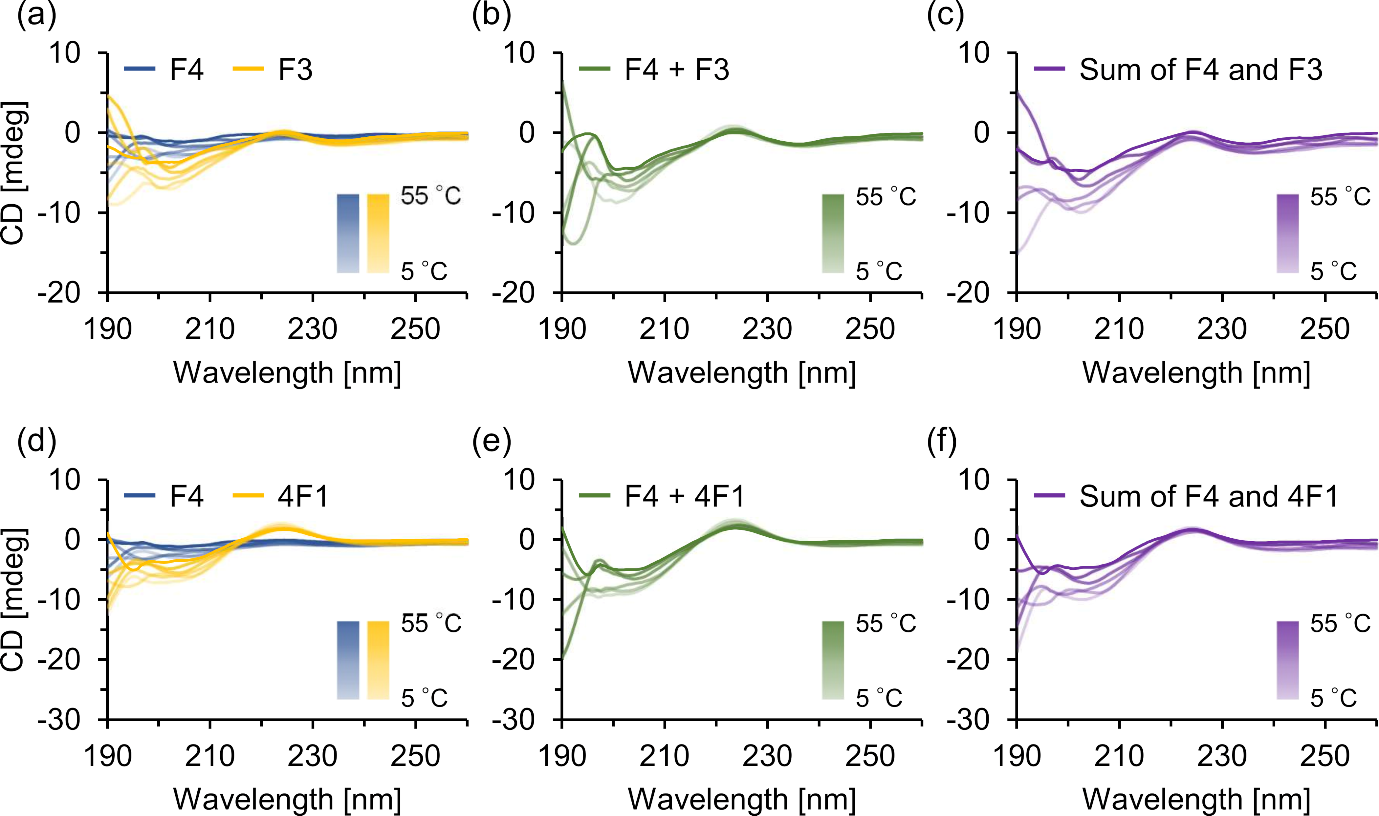


Figure S12. CD spectra upon heating shown in mdeg.

(a) single-component F4 (0.0210 mg/mL) and F3 (0.0790 mg/mL), (b) F4 + F3 (F4 0.0210 mg/mL and F3 0.0790 mg/mL), (c) Sum of the single-component F4 and F3, (d) single-component F4 (0.0210 mg/mL) and 4F1 (0.0508 mg/mL), (e) F4 + 4F1 (F4 0.0210 mg/mL and 4F1 0.0508 mg/mL), and (f) Sum of the single-component F4 and 4F1.


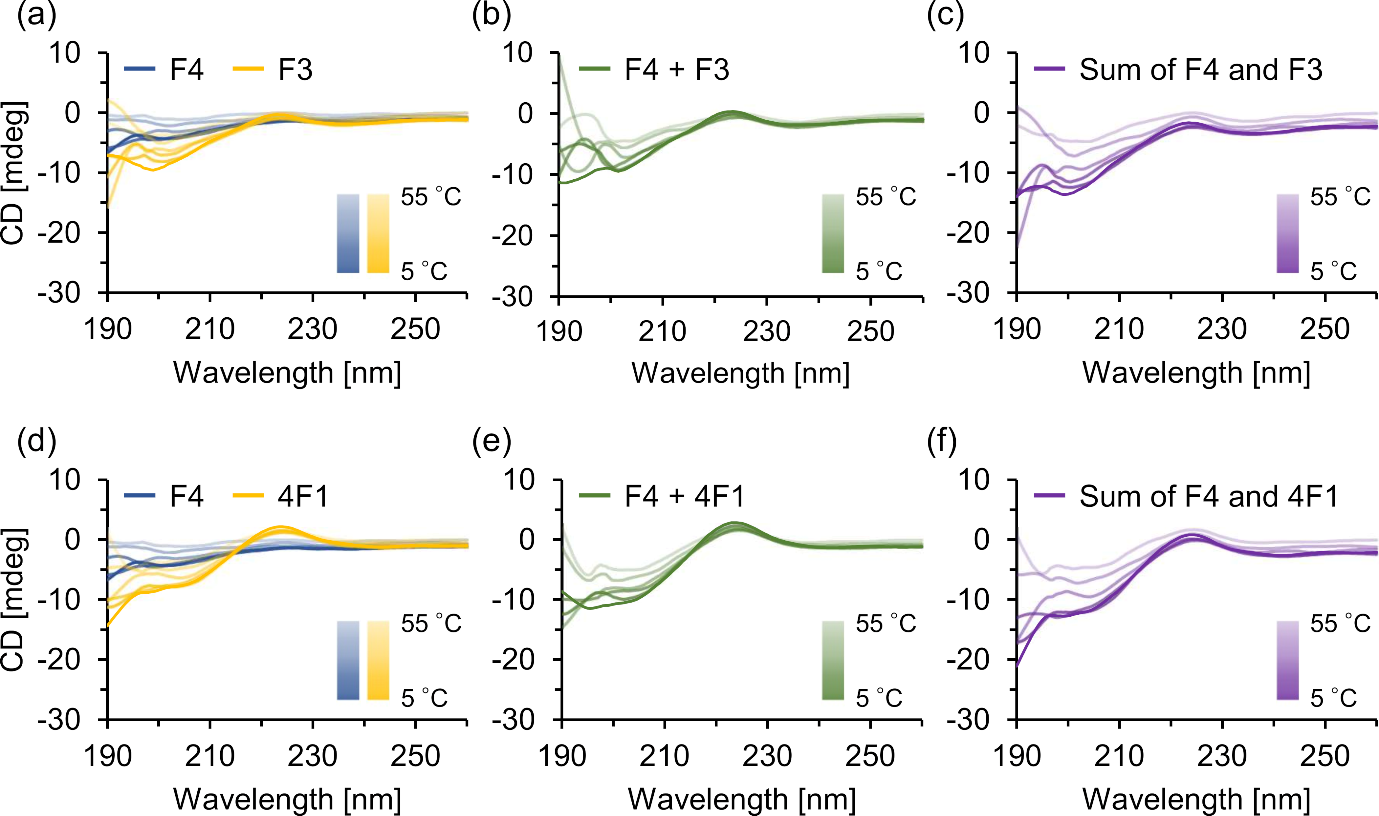


Figure S13. CD spectra upon cooling shown in mdeg.

(a) single-component F4 (0.0210 mg/mL) and F3 (0.0790 mg/mL), (b) F4 + F3 (F4 0.0210 mg/mL and F3 0.0790 mg/mL), (c) Sum of the single-component F4 and F3, (d) single-component F4 (0.0210 mg/mL) and 4F1 (0.0508 mg/mL), (e) F4 + 4F1 (F4 0.0210 mg/mL and 4F1 0.0508 mg/mL), and (f) Sum of the single-component F4 and 4F1.


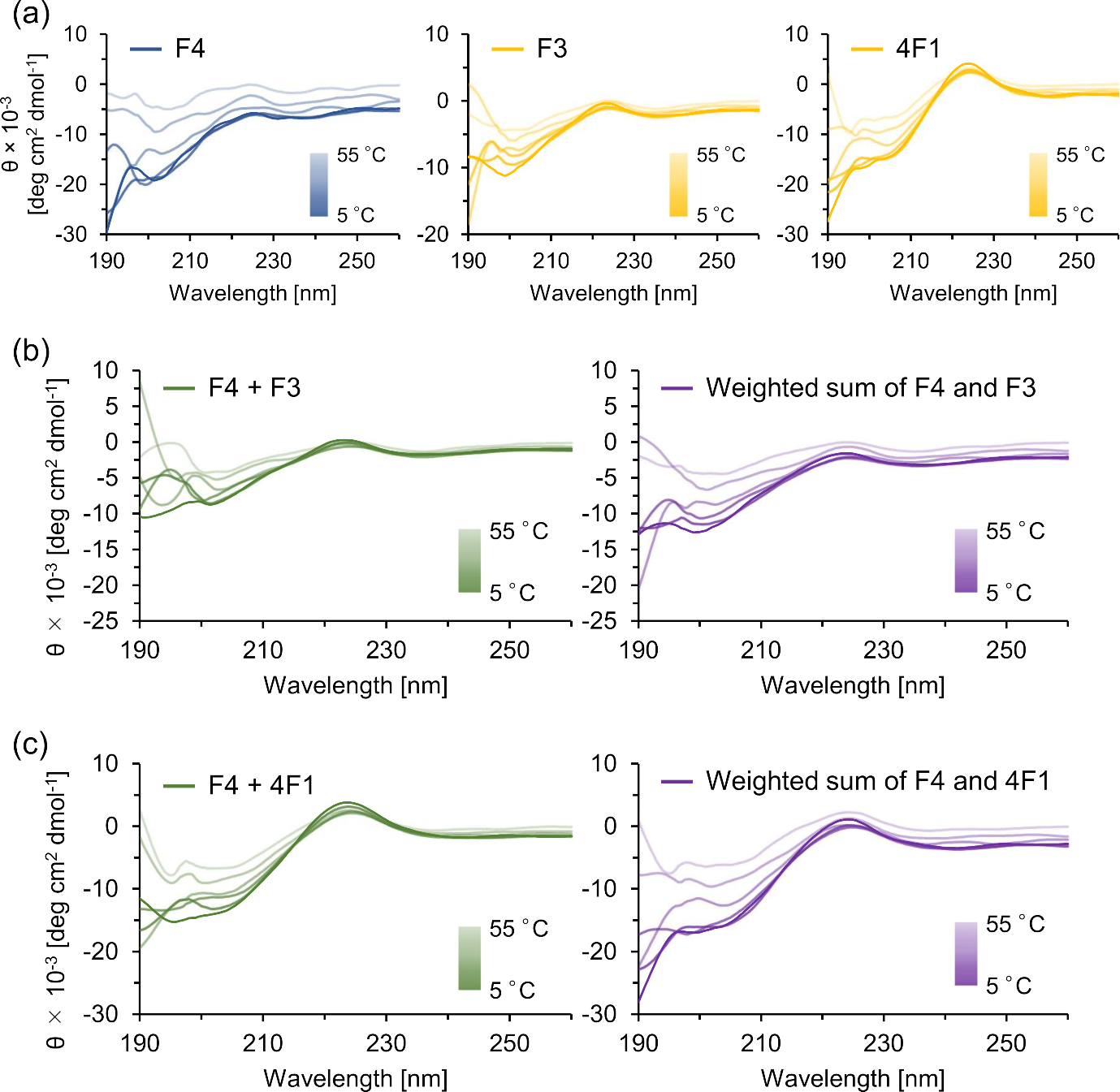


Figure S14. CD spectra upon cooling.

(a) F4 (0.0210 mg/mL, left), F3 (0.0790 mg/mL, middle), and 4F1 (0.0508 mg/mL, right), (b) F4 + F3 (F4 0.0210 mg/mL and F3 0.0790 mg/mL, left) and calculated spectra based on the single-component F4 and F3 (right), (c) F4 + 4F1 (F4 0.0210 mg/mL and 4F1 0.0508 mg/mL, left) and calculated spectra based on the single-component F4 and 4F1 (right).


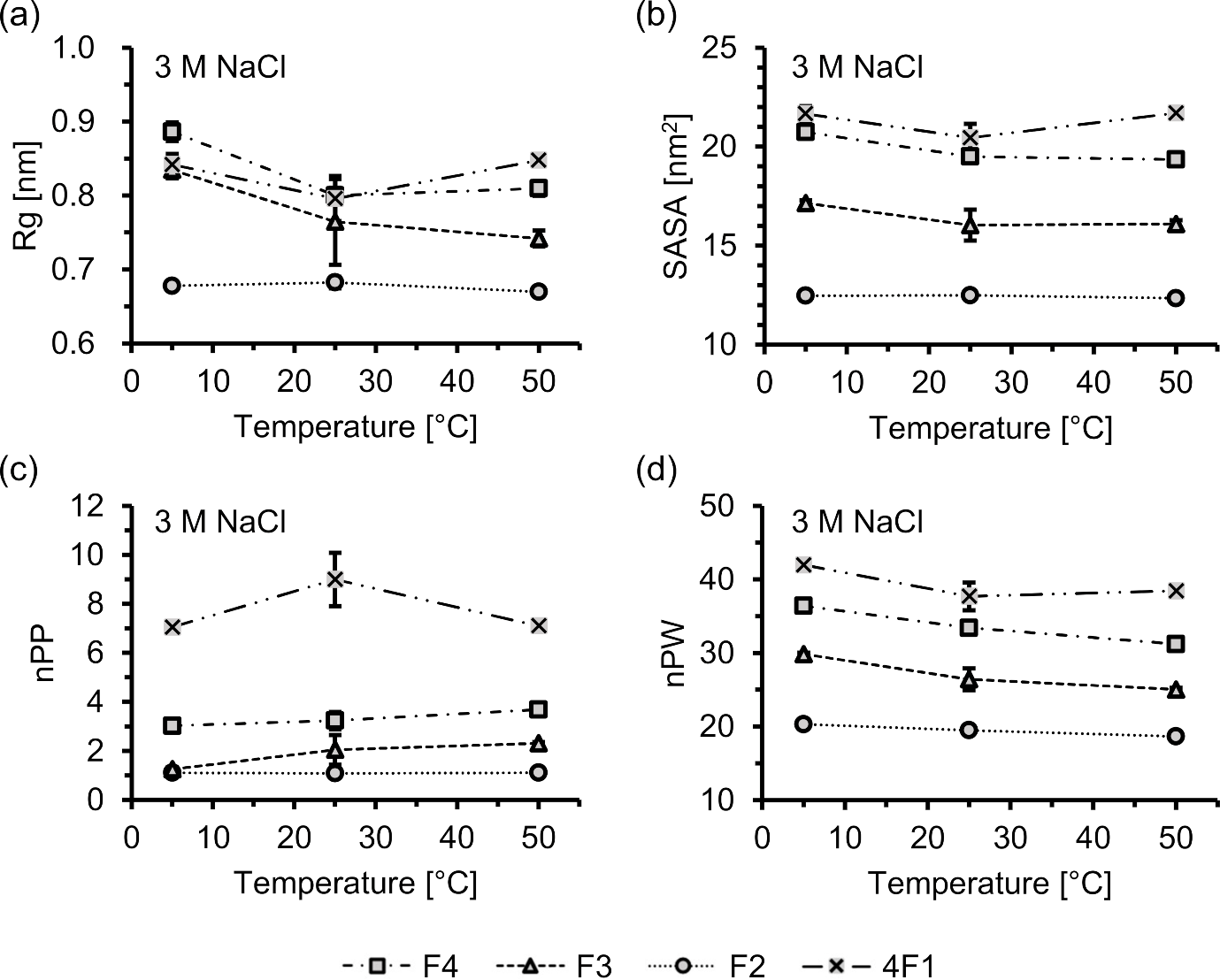


Figure S15. MD simulation under 3M NaCl.

(a) Radius of gyration (Rg), (b) solvent-accessible surface area (SASA), (c) number of intramolecular hydrogen bonds (nPP), and (d) number of hydrogen bonds between peptide and water molecules (nPW) for F4 (square), F3 (triangle), F2 (circle), and 4F1 (cross) with 3 M NaCl. Data represent the average of three independent simulation runs, with standard errors (SE) shown as error bars. Values obtained at 5 °C, 25 °C, and 50 °C are connected by lines for visual clarity.
